# Aberrant splicing of Ca_V_1.2 calcium channel induced by decreased Rbfox1 enhances arterial constriction during diabetic hyperglycemia

**DOI:** 10.1007/s00018-024-05198-z

**Published:** 2024-04-04

**Authors:** Wei Hou, Shumin Yin, Pengpeng Li, Ludan Zhang, Tiange Chen, Dongxia Qin, Atta Ul Mustafa, Caijie Liu, Miaomiao Song, Cheng Qiu, Xiaoqing Xiong, Juejin Wang

**Affiliations:** 1 Key Laboratory of Targeted Intervention of Cardiovascular Disease, Collaborative Innovation Center for Cardiovascular Disease Translational Medicine, Nanjing Medical University, Nanjing, Jiangsu China; 2 Department of Physiology, Nanjing Medical University, Nanjing, Jiangsu China; 3 The Affiliated Taizhou People’s Hospital of Nanjing Medical University, Taizhou School of Clinical Medicine, Nanjing Medical University, Taizhou, Jiangsu China; 4 Nanjing Comprehensive Stroke Center, Affiliated Nanjing Brain Hospital, Nanjing Medical University, Nanjing, Jiangsu China

**Keywords:** Alternative splicing, Arterial constriction, Ca_V_1.2 calcium channel, Diabetes, Hyperglycemia

## Abstract

Diabetic hyperglycemia induces dysfunctions of arterial smooth muscle, leading to diabetic vascular complications. The Ca_V_1.2 calcium channel is one primary pathway for Ca^2+^ influx, which initiates vasoconstriction. However, the long-term regulation mechanism(s) for vascular Ca_V_1.2 functions under hyperglycemic condition remains unknown. Here, Sprague–Dawley rats fed with high-fat diet in combination with low dose streptozotocin and Goto-Kakizaki (GK) rats were used as diabetic models. Isolated mesenteric arteries (MAs) and vascular smooth muscle cells (VSMCs) from rat models were used to assess K^+^-induced arterial constriction and Ca_V_1.2 channel functions using vascular myograph and whole-cell patch clamp, respectively. K^+^-induced vasoconstriction is persistently enhanced in the MAs from diabetic rats, and Ca_V_1.2 alternative spliced exon 9* is increased, while exon 33 is decreased in rat diabetic arteries. Furthermore, Ca_V_1.2 channels exhibit hyperpolarized current–voltage and activation curve in VSMCs from diabetic rats, which facilitates the channel function. Unexpectedly, the application of glycated serum (GS), mimicking advanced glycation end-products (AGEs), but not glucose, downregulates the expression of the splicing factor Rbfox1 in VSMCs. Moreover, GS application or Rbfox1 knockdown dynamically regulates alternative exons 9* and 33, leading to facilitated functions of Ca_V_1.2 channels in VSMCs and MAs. Notably, GS increases K^+^-induced intracellular calcium concentration of VSMCs and the vasoconstriction of MAs. These results reveal that AGEs, not glucose, long-termly regulates Ca_V_1.2 alternative splicing events by decreasing Rbfox1 expression, thereby enhancing channel functions and increasing vasoconstriction under diabetic hyperglycemia. This study identifies the specific molecular mechanism for enhanced vasoconstriction under hyperglycemia, providing a potential target for managing diabetic vascular complications.

**Supplementary Information:**

The online version contains supplementary material available at 10.1007/s00018-024-05198-z.

## Introduction

As a common metabolic disorder, diabetes mellitus affects 0.5 billion population, representing an increasing challenge to the health care around the world [1]. The most typical metabolic abnormality of diabetes is elevated blood glucose (hyperglycemia). A persistent hyperglycemic condition leads to the production of chemical moieties known as advanced glycation end-products (AGEs), which play a central role in the pathophysiology of diabetic cardiovascular complications [2], including hypertension, coronary heart disease and myocardial infarction [3, 4]. Hyperglycemia induces abnormal vasoconstriction and vasodilation [5], especially in small arteries, leading to the dysregulated blood pressure. Besides endothelial cells [6, 7], dysregulated functions of vascular smooth muscle cells (VSMCs) are known to play an important role in diabetic arteries in human and animals [8–10]. However, the long-term regulation mechanisms of VSMCs remain unclear.

The L-type Ca^2+^ channel Ca_V_1.2 is essential for arterial constriction, which responds to membrane potential and provides sustained influx of Ca^2+^ ions to activate vascular smooth muscle contraction [11]. Upon acute increases in extracellular glucose and in high fat diet (HFD)-induced diabetic hyperglycemia, PKA-mediated Ca_V_1.2 α_1C_ Ser1928 phosphorylation is correlated with enhanced Ca^2+^ influx in VSMCs and the reduction in arterial diameter and blood flow in vivo, which has been confirmed as one of key molecular signaling events underlying potentiation of Ca_V_1.2 channel activity and vasoconstriction [12–14]. However, compared with control rats, diabetic rats exhibited enhanced myogenic tone when exposed to a normal range of glucose concentration. Persistent hyperglycemic conditions induce aberrant genes transcription, contributing to the metabolic memory in cardiovascular dysfunction [15, 16]. Notably, optimal glycemia control cannot completely prevent the vascular complications in diabetic patients [17], and glycemic levels do not fully explain the association between long-term vascular complications and mortality in type 1 diabetes [18]. Therefore, besides the short-term effect induced by hyperglycemia, we speculated that there is an alternative long-term mechanism mediating the abnormal vascular Ca_V_1.2 channel function in diabetic arteries, leaving a knowledge gap.

Alternative splicing (AS), as one of post-transcriptional modulation mechanisms, is key to eukaryotic gene expression and cellular functions [19, 20]. Over 95% of human multi-exon genes are regulated by AS [21, 22]. The human Ca_V_1.2 pore-forming α_1C_ subunit-encoding gene *CACNA1C* has at least 20 alternatively spliced exons [23]. Among them, we have previously demonstrated the unique roles for 3 common spliced exons in regulating the electrophysiological and pharmacological functions of the cardiovascular Ca_V_1.2 channel, including the mutually exclusive exon 8/8a, cassette exon 9* and exon 33 [24–27]. Under pathological conditions, the splicing patterns of Ca_V_1.2 α_1C_ subunit can be altered. For example, in rat [26] and human [27] hypertrophic hearts, the expressions of Ca_V_1.2 alternative exon 9* and exon 33 were upregulated, respectively. Furthermore, in hypertensive arteries, Ca_V_1.2 alternative exon 8a and exon 9* increased, while alternative exon 33 decreased [24, 28]. However, it remains unknown whether the splicing pattern of Ca_V_1.2 channel is altered in diabetic arteries.

As an RNA-binding protein, the splicing factor regulates different splicing events by binding to the motifs of pre-mRNAs. *CACNA1C* has several binding sequences for different splicing factors, such as PTBP1 and Rbfox1/2 proteins [29]. Rbfox proteins are known to bind to the element of UGCAUG in pre-mRNA to regulate the splicing events [30, 31]. Through bioinformatic analysis, it has been discovered that *CACNA1C* gene contains multiple UGCAUG elements, which can be bound by Rbfox1/2 to regulate its AS events during neuronal development [32]. Previously, we found that Rbfox1/2 dynamically regulates Ca_V_1.2 exons 9* and 33 in VSMCs, playing important roles in vasoconstriction of hypertensive arteries [28, 33]. However, the roles of Rbfox proteins in modulating the key function of vascular Ca_V_1.2 channels and arterial constriction during diabetic hyperglycemia remain unidentified.

In this work, we hypothesize that the downregulation of Rbfox1 in diabetic arteries leads to aberrant splicing of vascular Ca_V_1.2 channel. This, in turn, results in persistently enhanced vasoconstriction during diabetic hyperglycemia. Thus, we have uncovered the long-term mechanism that mediates the function of vascular Ca_V_1.2 channels and the persistently enhanced vasoconstriction of small resistance arteries during hyperglycemia.

## Materials and methods

### Animal model and human samples

**Fig. 1:**
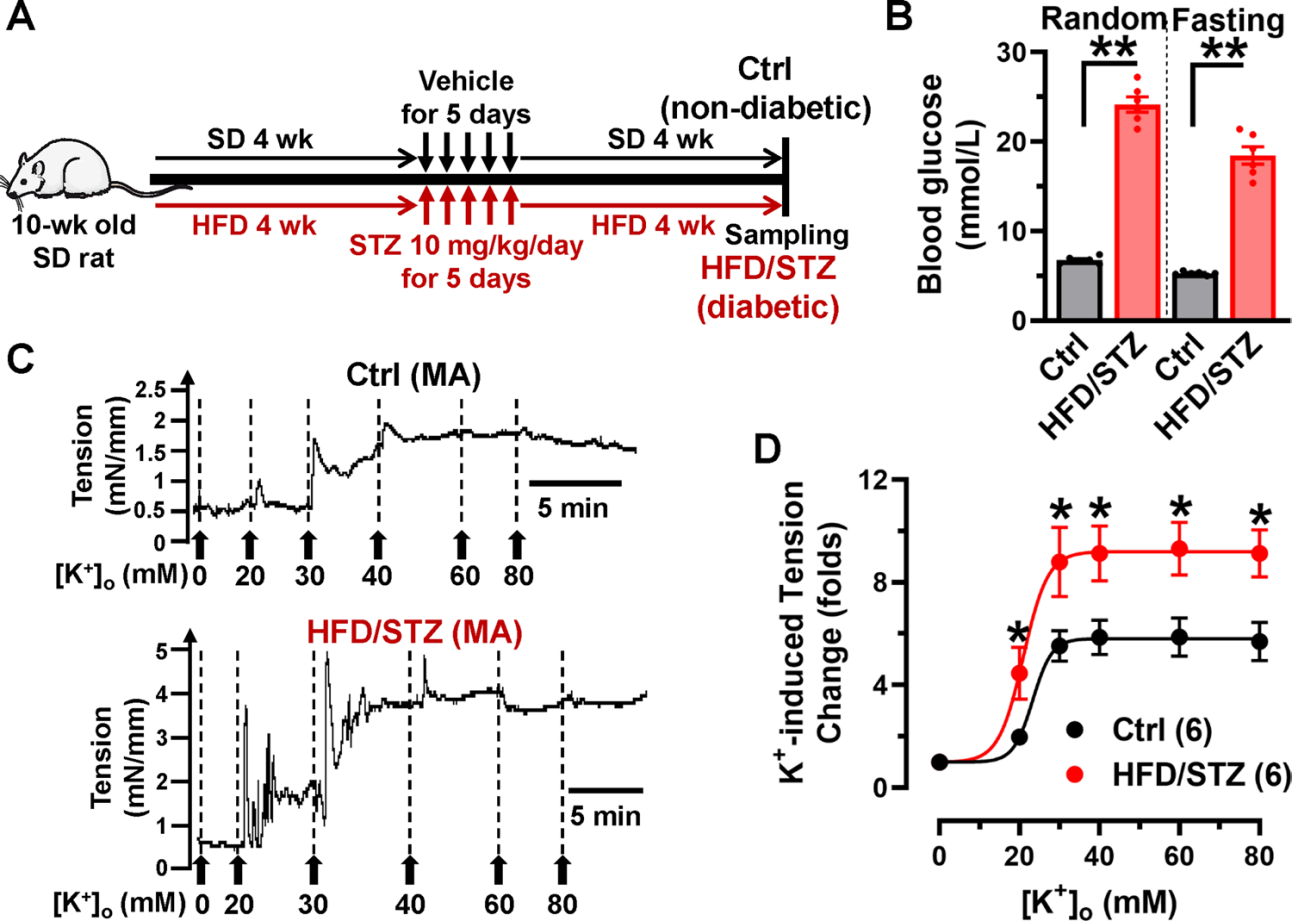
K^+^‐induced vasoconstriction is increased in mesenteric arteries of diabetic rats. **A** Diabetic rat models were established using a combination of HFD and STZ. Briefly, 10-week-old rats were fed a HFD for 4 weeks before a low dose of STZ was administered for 5 consecutive days, followed by another 4 weeks of HFD. Control rats were fed a standard diet and administrated with a vehicle. **B** Random and fasting blood glucose levels were monitored in the rats. ** *P* < 0.01, 1-way ANOVA followed by a Tukey’s post hoc test. *n* represents the number of rats. **C** Traces of mesenteric artery (MA) tension in response to increased KCl extracellular concentration (from 0 to 80 mmol/L KCl) in nondiabetic and diabetic MAs are shown. **D** Plots of concentration-tension relationship were represented and fitted with Boltzmann equation for the arteries from control (*n* = 16 arteries from 6 rats) or HFD/STZ-treated rats (*n* = 18 arteries from 6 rats). * *P* < 0.05 vs. control rats, 2-way ANOVA followed by Sidak’s multiple comparisons

All animal experiments were approved by the Committee on Animal Care of Nanjing Medical University and were conducted according to the NIH Guidelines for the Care and Use of Laboratory Animals. All studies involved with animals were reported in accordance with the ARRIVE guidelines. Male Sprague–Dawley (SD) rats (≈180 g) and Wistar rats used in this study were purchased from Jiangsu Laboratory Animal Center (Nanjing). Goto-Kakizaki (GK) rats (16-weeks-old) were purchased from Gene and Peace Biotechnology Ltd (Yangzhou, China). The diabetic rat models were established with a HFD in combination with a low dose of streptozotocin (STZ). Briefly, the 10-weeks-old rats were fed with HFD (45%) for 4 weeks before administration of STZ (intraperitoneal injection, 10 mg/kg/day, in 0.1 mol/L citric acid-sodium citrate buffer solution, pH 4.5) for 5 successive days (Fig. 1A), This was followed by feeding with HFD for another 4 weeks. Control rats were fed a standard diet and injected with an equal volume of citric acid-sodium citrate buffer solution. HFD/STZ-treated rats and GK rats with a random blood glucose level of ≥16.7 mmol/L were considered diabetic [34] and included for further analysis. Human cerebral arteries were collected from patients undergoing surgeries at the Affiliated Brain Hospital of Nanjing Medical University. The study was approved by the Institutional Review Board of Nanjing Medical University (Reference No. 2022/550), and each patient signed a written informed consent document. The clinical characteristics of the patients were listed in Table S1 in the Supplementary Information.

### Preparation of glycated serum

Glycated serum (GS) was used to mimic the AGEs [35, 36]. Briefly, GS was prepared by incubating fetal bovine serum (FBS) (HyClone) and d-glucose (90 g/L) at 37 °C for 3 weeks after filtering and sterilizing. Non-glycated serum (NG) was prepared using the same protocol without adding d-glucose. The concentrations of AGEs in GS were determined by an ELISA assay (Jiancheng Bioengineering Institute, Nanjing, China), which was 84.82 ng/mL in GS and 26.02 ng/mL in NG, respectively. Additionally, the concentration of AGEs was 47.53 ng/mL in the serum from HFD/STZ-treated rats.

### Measurement of vascular constriction

The Myograph system (620M, DMT, Denmark) was used to evaluate vasoconstriction by measuring the isometric tension [33]. Briefly, second-order mesenteric arteries (MAs), were isolated in K^+^-free Hanks’ solution (containing in mmol/L: 1.26 CaCl_2_, 0.41 MgSO_4_-7H_2_O, 137.93 NaCl, 0.34 Na_2_HPO_4_, 0.49 MgCl_2_-6H_2_O, 4.17 NaHCO_3_, 5.56 d-Glucose). After removing connective tissues, the arteries were cut into 1-to-1.2-mm segments, then mounted with 20-μm wires and set at a resting tension of 0.1 g. All segments were equilibrated for 45 min with intermittent washes every 15 min using normal Hanks’ solution. We used 60 mmol/L KCl (dissolved in K^+^-free Hanks’ solution) to perfuse the mounted arteries and check their vascular contractility. After that, the chamber was perfused with normal saline solution. Vasoconstriction was then measured by stepwise increasing extracellular KCl concentration from 20 to 80 mmol/L. The change in vascular constriction (folds) was calculated as the vascular tension under K^+^-containing bath solution divided by the resting tension. The dosage-effect curves were fitted with the Boltzmann equation. The Ca^2+^ ionophore ionomycin was used at the end of experiment to induce the maximal contractility and for quality control. Arteries that did not achieve a 10-folds tension change were excluded for further analysis. To check the vasoconstriction of MAs from control or HFD/STZ-treated rats, we only used 2–3 arteries from each rat to measure their vascular contractility.

### Isolation of VSMCs

Enzymatic isolation of VSMCs was carried out as previously described [28, 33]. Briefly, rats’ aorta or MAs were isolated rapidly and placed in 4 °C in Hanks’ solution. After cutting into segments, the arteries were digested in 2 steps. First, the tissues were incubated in a solution contained 1 mg/mL albumin, 1 mg/mL papain, and 2 mg/mL dithioerythritol, followed by digestion in a solution contained 1 mg/mL albumin, 5 mg/mL collagenase II, and 1 mg/mL hyaluronidase for 30 min each step. Isolated cells were cultured in DMEM (Gibco) medium and replaced with fresh medium the next day. We only used primary isolated VSMCs for the experiments.

### Cell culture and treatments

Isolated VSMCs were cultured in DMEM (Gibco) medium with 10% FBS under a 5% CO_2_ incubator at 37℃. GS (20%), methylglyoxal (MGO) (500 μmol/L), or mannitol (25 mmol/L) was used to treat VSMCs for 48–72 h. For cell transfection experiments, VSMCs were seeded in 6-well cell culture plates at a density of 60–70%. Then, 50 nmol/L pooled siRNAs (RiboBio, Guangzhou, China) or 2 μg expression plasmids were transfected into cells using the Lipofectamine 3000 kit (Invitrogen) and cultured for another 48 h. The sequence information of siRNAs targeting rat Rbfox1 mRNAs is listed in Table S2.

### Reverse-transcription polymerase chain reaction (RT-PCR)

Total RNA was extracted from arteries or VSMCs using Trizol reagent (Invitrogen). 2 μg of total RNA was reverse transcribed into cDNA using SuperScript III reverse transcriptase (Invitrogen). The primer sequences used in the present study are listed in Table S3. The gel images were captured using a Gel Imaging System (Tanon, Shanghai).

### Western blotting

Total protein was extracted from VSMCs or arterial tissues in RIPA lysis buffer with 1% phenylmethylsulfonyl fluoride. The protein concentration was determined by the Bradford assay. Protein samples were boiled in 5× SDS-sample buffer at 100℃ for 5 min. Equal amounts of the protein samples were separated by 10% SDS-PAGE and then transferred to a PVDF membrane (Millipore, 0.2 μm) under a constant voltage. A solution of 5% milk powder dissolved in *Tris*-buffered saline with Tween-20 (TBST) was used to block the membrane. The membranes were incubated with primary antibodies overnight at 4℃and then incubated with a horseradish peroxidase-linked secondary antibody (1:10,000) for 1 h at room temperature. Primary antibodies were not reused, and dilutions of the secondary antibody were maintained at 4 °C and reused up to three times. The information of the antibodies was listed in Table S4. The protein bands were visualized using the Immobilon Western Chemiluminescent HRP Substrate Kit (Millipore). All uncropped images of Western blotting were presented in Supplementary Information.

### Immunofluorescent staining

The localization of Rbfox1 in cells was determined through immunofluorescence staining. Briefly, freshly isolated MAs were fixed in 4% paraformaldehyde for 24 h at room temperature and then immersed in 15% sucrose and 30% sucrose for 10 min each step. Using a cryostation, the tissues were cut into 20-μm thickness. After permeabilizing with 0.2% Triton X-100 in PBS for 20 min, cells or tissues were blocked with 2% BSA in PBS for 40 min at room temperature. They were then incubated with rabbit anti-Rbfox1 (1 μg/mL, sc-135476, Santa Cruz Biotechnology) and mouse anti-α-smooth muscle actin (SMA) (0.4 μg/mL, NBP2-33006, Novus Biologicals) antibodies dissolved in the blocking solution at room temperature for 12 h. The information of antibodies was listed in Table S4. After washing with PBS, cells or tissues were incubated for 1 h with secondary antibodies labeled with Alexa Fluor 488 or 594 (R37114 or R37119, Molecular Probes) at room temperature. A confocal laser scanning microscope (LSM710, Carl Zeiss, Germany) was used to capture the fluorescence signal.

### Introduction of Rbfox1 expression plasmid or siRNAs into rat MAs

Rbfox1 plasmid or siRNAs were introduced into the MAs by a reversible permeabilization (RP) procedure as previously described [25]. First, MAs were incubated for 20 min at 4 °C in a solution contained (in mmol/L) 120 KCl, 2 MgCl_2_, 10 EGTA, 5 ATP, and 20 TES (pH 6.8). After that, the arteries were incubated in a solution containing 4 μg/mL Rbfox1 plasmid or 40 nmol/L siRNAs, 120 KCl, 2 MgCl_2_, 5 ATP, and 20 TES (pH 6.8) for 3 h at 4 °C and then in the same solution but with an elevated 10 MgCl_2_ for 30 min. Permeabilization was reversed by incubating the arteries for 30 min at room temperature in a physiological solution containing (in mmol/L) 140 NaCl, 5 KCl, 10 MgCl_2_, 5 glucose, and 2 MOPS (pH 7.1). The calcium concentration was gradually increased from 0 to 0.01, 0.1, and 1.8 mmol/L over a period of 45 min. The arteries were cultured in serum-free DMEM-F12 medium (Invitrogen) under 5% CO_2_ at 37 °C, and their endogenous expression of Rbfox1 was checked by Western blotting after 72 h of culture.

### Whole-cell recording of Ca_V_1.2 calcium channel currents

Whole-cell currents were recorded using an Axon 200B amplifier (Molecular Device, Sunnyvale, CA) and the pCLAMP software (version 10.7, Axon Instruments). The pipette solution contained (in mmol/L): 130 CsCl, 5 EGTA, 1 MgCl_2_, 10 HEPES, 2 Mg-ATP, 0.5 GTP, and 10 d-glucose. The pH was adjusted to 7.2, and the osmolarity was maintained at 290 to 300 mOsm. The bath solution contained (in mmol/L): 122 or 132 TEA-Cl, 10 HEPES, 1 MgCl_2_, 20 or 10 BaCl_2_, and 10 d-glucose. The pH was adjusted to 7.2, and the osmolarity was maintained at 300 to 310 mOsm.

The cells were held at −70 mV and then stepped from −50 to +50 mV. The currents were filtered at 1 to 5 kHz and sampled at 5 to 50 kHz, and the series resistance was <5 MΩ after >70% compensation. The P/4 protocol was used to subtract online the leak and capacitive transients. Current size was normalized to cell capacitance (*C*_m_), which was presented as current density. The data were not included if the leaky currents were >100 pA in VSMCs. GS or MGO were applied to the VSMCs for 24–48 h before the recording, and H-89 was added to VSMCs for 4 h prior to the experiments. The current–voltage (*I*–*V*) relationship curve was fitted with the equation: *I* = *G*_max_(*V* − *E*_rev_)/(1 + exp[*V* – *V*_0.5_]/*k*), where *G*_max_ represents the maximum conductance, *V* is testing potential, *E*_rev_ is the reversal potential, *V*_0.5_ is the half-activation potential, and *k* is the slope rate. The steady-state activation (SSA) curve was derived from an *I*–*V* protocol and calculated using the equation *G* = *I*/(*V* – *E*_rev_), where *G* represents the conductance. The curve was fitted with Boltzmann equation. The steady-state inactivation (SSI) curves were obtained from each test pulse, which were normalized to the maximal current amplitude of the normalizing pulse. The *SSI* curve were fitted with a single Boltzmann equation: *I*_relative_ = *I*_min_ + (*I*_max_ − *I*_min_)/(1 + exp(*V*_0.5,inact_ − *V*)/*k*), where *I*_relative_ represents the normalized current; *V*_0.5,inact_ is the potential for half-inactivation, and *k* is the slope value.

### Measurement of cytosolic Ca^2+^ concentration

Cytosolic calcium concentration was measured using 4 μmol/L Fluo-4 AM (Molecular Probes). VSMCs should be bathed in Tyrode solution and then transferred to laminin-coated culture dishes prior to imaging on the stage of a confocal microscope (LSM710, Carl Zeiss, German). The images were acquired in time series scanning mode and sampled at 1 fps, and the fluorescence intensity was analyzed offline. ​To calibrate the fluorescence signal of the intracellular Ca^2+^ indicator, we used the equation: Δ[Ca^2+^]_*i*_ = Δ*F*/*F*_0_ = (*F* − *F*_0_)/*F*_0_, where *F* is the fluorescence signal at any given time and *F*_0_ is the mean resting fluorescence, to calculate changes in intracellular calcium concentration.

### Statistical analysis

Data are expressed as means ± SEM. The “*n*” number represents the number of animals in Western blotting, RT-PCR experiments, and arteries during vascular function measurement. It also represents the number of cells in patch clamp study and fluorescence detection from at least 3 independent experiments. Statistical significance was analyzed using Student’s unpaired *t* test, 1-way ANOVA followed by a Tukey’s post hoc test, or 2-way ANOVA followed by Sidak’s multiple comparisons. *P* < 0.05 was considered a significant difference.

## Results

### Hyperglycemia leads to persistently enhanced K^+^-induced vasoconstriction in diabetic arteries

For the HFD/STZ-induced diabetic model, we used a combination treatment of HFD and STZ to generate the diabetic rat model for ≈9 weeks (Fig. 1A). The peripheral blood was collected to measure the blood glucose levels in HFD/STZ and GK diabetic rats (type 2 diabetic model). Compared to control rats, diabetic rats exhibited significant elevated random and fasting blood glucose levels (Fig. 1B; Fig. S1A), indicating that hyperglycemia was well-established in the diabetic rat models. To determine the vascular tension of the arteries, MAs were isolated from the rats and perfused with K^+^-free Hanks’ solution. They were then exposed to increasing KCl concentrations to induce vasoconstriction ex vivo (Fig. 1C; Fig. S1C). In comparison to control rats, K^+^-induced vasoconstriction was significantly increased in the arteries from HFD/STZ-treated rats (Fig. 1D) and GK rats (Fig. S1D), indicating that isolated diabetic arteries have persistently enhanced vasoconstriction. Of significance, the calcium channel blocker (CCB) nifedipine-induced vasodilation was greater in the MAs from diabetic rats compared to those from control rats, indicating that diabetic arteries are more sensitive to CCB (Fig. S2).

### Facilitated activation of Ca_V_1.2 channel in VSMCs from diabetic arteries

**Fig. 2:**
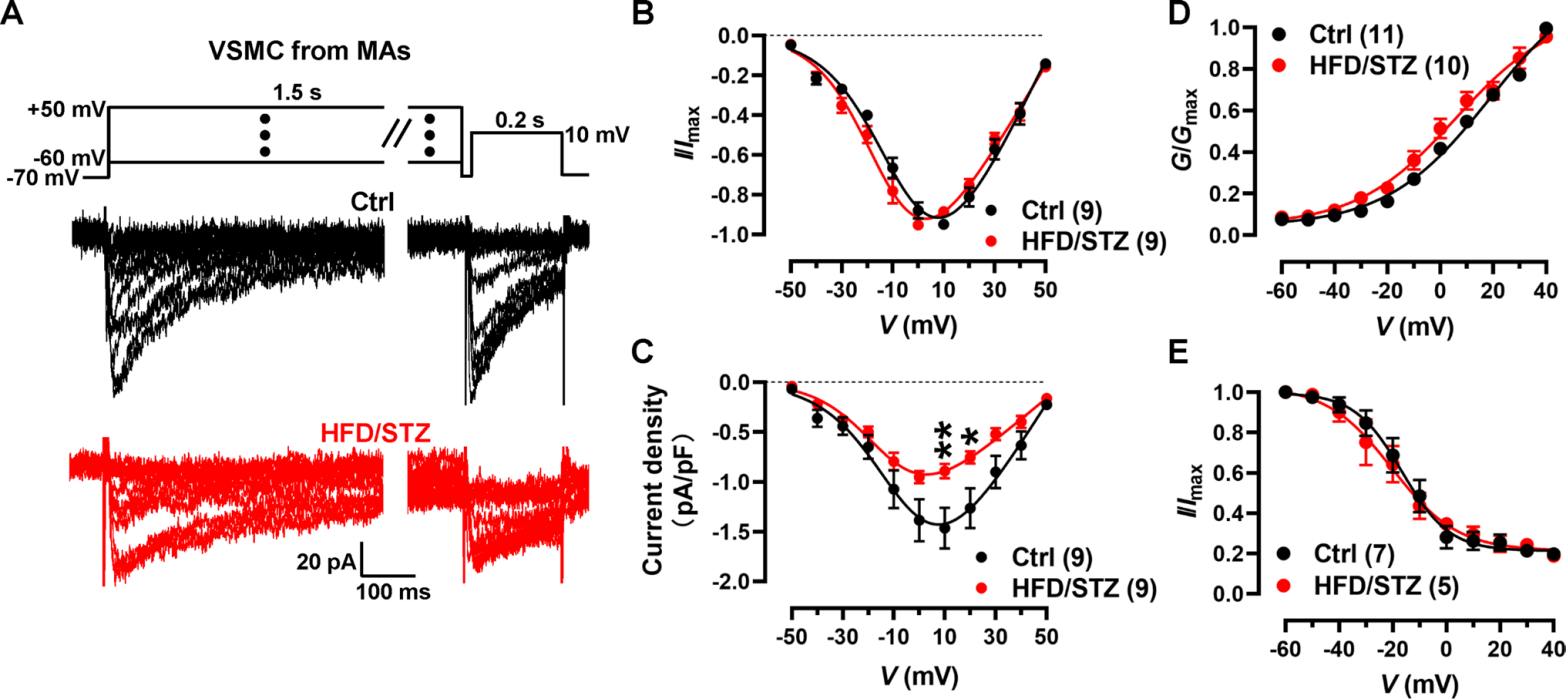
Ca_V_1.2 channel activation curve is left-ward shifted (hyperpolarized) in isolated VSMCs from diabetic rats. **A** VSMCs were freshly isolated from mesenteric arteries from either control or HFD/STZ-treated rats. Ca_V_1.2 channel currents were recorded under various testing potentials, ranging from − 50 to 50 mV over 1500 ms. Subsequently, the currents were recorded by a 200 ms test pulse at 10 mV in a 20 mmol/L Ba^2+^ bath solution, while holding the cells at − 70 mV. **B** Plots of Ca_V_1.2 *I*–*V* curve of VSMCs were analyzed and fitted with the equation. **C** Current densities of Ca_V_1.2 channel were calculated by dividing the currents by the cell capacitance (*C*_m_) of the VSMCs from either control or HFD/STZ-treated rats. * *P* < 0.05, ** *P* < 0.01, 2-way ANOVA followed by Sidak’s multiple comparisons. **D** Plots of SSA curves of Ca_V_1.2 channel in VSMCs from either control or HFD/STZ-treated rats were derived from recordings by *I*–*V* protocol. **E** Plots of SSI curves of Ca_V_1.2 channel in VSMCs were recorded by *SSI* protocol and fitted with the Boltzmann equation. *n* represents the number of cells from at least 3 rats for each group

Given that Ca^2+^ influx from Ca_V_1.2 channels in VSMCs is key for vasoconstriction [11], we next investigated the electrophysiological properties of the Ca_V_1.2 channel in VSMCs during diabetic hyperglycemia. Previously, we performed the experiments using 2 concentrations of nifedipine to assess the levels of expression of Ca_V_1.2 and Ca_V_1.3 currents based on their different sensitivities to dihydropyridine. The results showed that nifedipine inhibits calcium currents similarly at concentrations of 1 μmol/L or 10 μmol/L [25], indicating that the major Ca^2+^ currents in VSMCs are mediated by Ca_V_1.2, not the Ca_V_1.3 channels. Here, whole-cell patch clamp was applied to record Ca_V_1.2 currents in isolated VSMCs from rats MAs (Fig. 2A). As shown in Fig. 2B, the normalized *I*–*V* curve of diabetic VSMCs shifted towards the left (hyperpolarized) by approximately 7.4-mV in *V*_0.5_ (half-activation potential) compared to nondiabetic cells. However, the cell capacitance of VSMCs was similar between control and HFD/STZ-treated rats (Fig. S3A). The current densities of Ca_V_1.2 channels in the VSMCs from diabetic rats were lower than those from control rats (Fig. 2C), which may be attributed to diabetic dyslipidemia [37].

To further confirm the detailed electrophysiological properties of Ca_V_1.2 in diabetic rats, we checked the SSA and SSI curves, respectively. As shown in Fig. 2D, the activation curve and *V*_0.5,act_ of Ca_V_1.2 channel in the VSMCs from diabetic models were shifted towards the left (Table S5), but the inactivation curve (Fig. 2E) and *V*_0.5,inact_ (half-inactivation potential) remained similar (Table S5). These data suggested that diabetic hyperglycemia facilitates the activation of vascular Ca_V_1.2 channels upon depolarization from the resting membrane potential.

### Aberrant splicing of Ca_V_1.2 channel are presented in the arteries of diabetic rats and patients

**Fig. 3:**
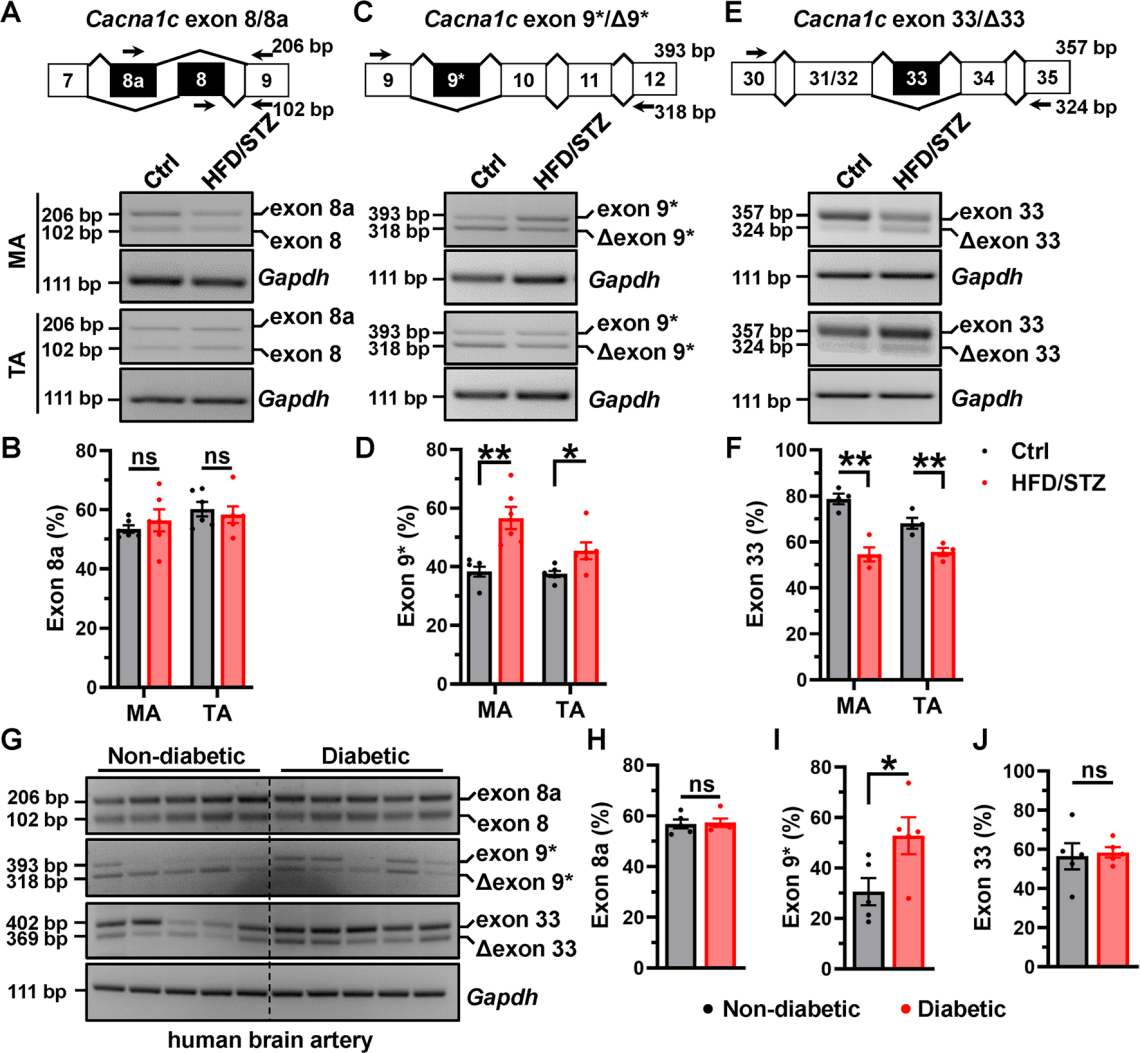
Ca_V_1.2 channel is aberrantly spliced in the arteries from diabetic rats. **A** Specific primers were used to amplify and detect rat *Cacna1c* mRNA with alternative exon 8/8a, with the upper bands showing the inclusion of exon 8a, and the low bands indicating exon 8. Mesenteric arteries (MAs) and thoracic arteries (TAs) from control or HFD/STZ-treated rats were collected to perform RT-PCR. *Gapdh* mRNA was detected as loading control. **B** Combined data showed the proportions of Ca_V_1.2 with alternative exon 8a in MAs or TAs, respectively. **C** The primers were used to detect rat *Cacna1c* mRNA with alternative exon 9*, with the upper and low bands showing the inclusion or exclusion of exon 9*, respectively. **D** The data are presented as bar chart to show the proportions of Ca_V_1.2 with alternative exon 9* in MAs or TAs, respectively. **E** The specific primers were also used to detect rat *Cacna1c* mRNA with alternative exon 33. **F** The data are shown as bar charts to show the proportions of Ca_V_1.2 with alternative exon 33 in mesenteric arteries or thoracic arteries, respectively. * *P* < 0.05, ** *P* < 0.01 vs control, unpaired *t* test. ns indicates no significant differences. *n* represents the number of rats. **G** The specific primers were used to detect *Cacna1c* mRNA with alternative exons. The data are presented as bar charts to show the proportions of Ca_V_1.2 with alternative exon 8a (**H**), exon 9* (**I**) or exon 33 (**J**) in cerebral arteries from the patients with or without diabetes. The ratios between the upper band and the sum of the upper and lower bands present the proportions of Ca_V_1.2 channel with exon 8a, exon 9* or exon 33. * *P* < 0.05 vs non-diabetic patients, unpaired *t* test. ns indicates no significant differences. *n* represents the number of patients

The splicing pattern of the vascular Ca_V_1.2 channel can be altered under pathological conditions such as atherosclerosis [38] and hypertension [24, 28, 33]. However, it is unknown whether it changes under diabetic hyperglycemia. Hence, we collected the MAs and thoracic arteries (TAs) from nondiabetic and diabetic rats. Using the RT-PCR approach, we found that the proportion of Ca_V_1.2 channels with exon 8a (Ca_V_1.2_E8a_) did not change in the arteries (Fig. 3A, B). However, the proportions of Ca_V_1.2 channels with exon 9* (Ca_V_1.2_E9*_) in the arteries from diabetic rats were significantly increased by 18.3% in MAs and 7.9% in TAs compared to those in nondiabetic arteries, respectively (Fig. 3C, D). Particularly, Ca_V_1.2 channels with exon 33 (Ca_V_1.2_E33_) decreased by 24.2% in MAs and 12.5% in TAs compared to those from nondiabetic rats (Fig. 3E, F). Exceptionally, we observed a more robust change of AS events in MAs than in TAs from rats, indicating that AS of Ca_V_1.2 in the diabetic artery mainly occurs in the small arteries, which are resistance vessels for arterial blood pressure regulation.

Based on our study using the GK rat as a type 2 diabetic model, we observed a significant increase in the proportion of Ca_V_1.2_E9*_ channel and a decrease in Ca_V_1.2_E33_ channel (Fig. S4A–D). Importantly, we collected the arteries from the non-hypertensive HFD/STZ rats and examined the expressions of Ca_V_1.2 alternative exons 9* and 33. The results confirmed that the expression of Ca_V_1.2_E9*_ channel was increased, while Ca_V_1.2_E33_ was decreased (Fig. S4F–I). These findings suggest that hyperglycemia, independent of hypertension, can directly induce aberrant splicing of the Ca_V_1.2 channel in the arteries.

Furthermore, we collected arterial tissues from both diabetic and non-diabetic patients, and found that the proportion of Ca_V_1.2_E9*_ channel was significantly increased in the arteries of diabetic patients. However, the proportion of Ca_V_1.2_E8a_ and Ca_V_1.2_E33_ remained unchanged (Fig. 3G–I). These results suggest that splice-variants of Ca_V_1.2 channels are aberrantly expressed in diabetic arteries, potentially leading to irregular functions of the Ca_V_1.2 channel.

### Expression of Rbfox1 is decreased in diabetic arteries

**Fig. 4:**
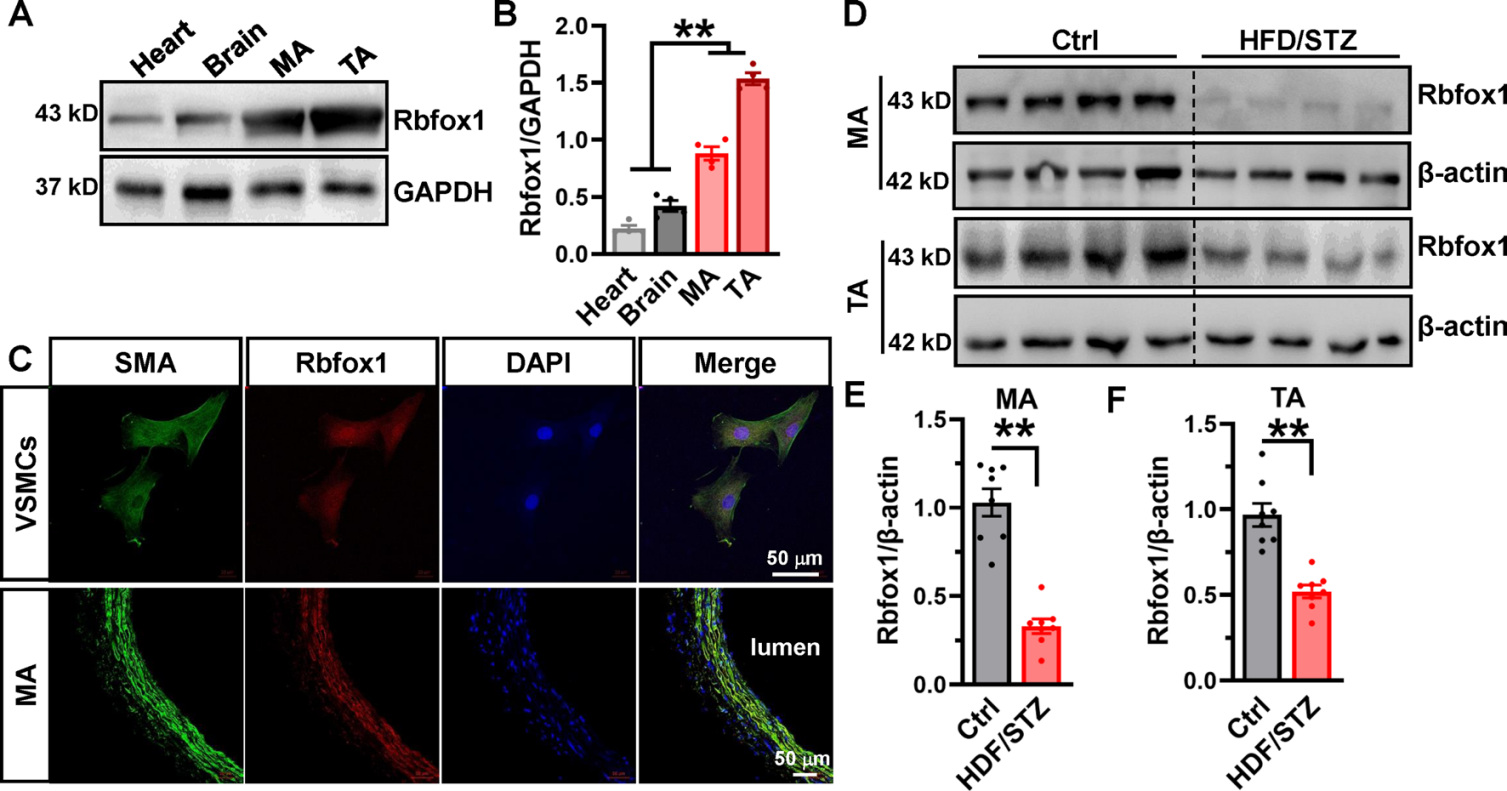
Rbfox1 is abundantly expressed in the arterial tissues, but it is downregulated in the arteries from diabetic rats. **A** Rbfox1 protein was detected via Western blotting in different tissues from rats, with the GAPDH protein serving as an internal control. **B** Expression levels of Rbfox1 protein were analyzed and presented as a bar chart, ** *P* < 0.01, 1-way ANOVA followed by a Tukey’s post hoc test. **C** Immunofluorescence staining was used to determine the localization of Rbfox1 in smooth muscles using anti-Rbfox1 and anti-α-smooth muscle actin in isolated VSMCs or mesenteric arteries (MAs) from rats; the right panel shows the merged images. **D** MAs and thoracic arteries (TAs) from control or HFD/STZ-treated rats were isolated to determine the expression level of Rbfox1 protein by Western blotting. β-actin was detected as a loading control. The relative Rbfox1 expression levels in the MAs (**E**) and TAs (**F**) were analyzed and presented as bar charts comparing HFD/STZ-treated rats to control rats. ** *P* < 0.01, unpaired *t* test. *n* represents the number of rats

Rbfox1 is known as a splicing factor that regulates the Ca_V_1.2 alternative exons 9* and 33 during neural development [32], and has also been found to regulate Ca_V_1.2 AS events in hypertensive arteries [33]. To assess the role of Rbfox1 in diabetic arteries, we used Western blotting to detect the expressing levels of Rbfox1 in different rat tissues. We found that Rbfox1 is highly expressed in arterial tissues, including MAs and TAs, compared to brain and cardiac tissues (Fig. 4A, B). Immunofluorescence staining confirmed that Rbfox1 is mainly located in the nucleus of isolated VSMCs and arterial smooth muscle, as stained with α-SMA (Fig. 4C), suggesting that Rbfox1 is an RNA-binding protein. Next, the MAs and TAs of nondiabetic or diabetic rat models were isolated, and Western blotting showed that the expressions of Rbfox1 are remarkably decreased in the arteries from diabetic rat models compared to nondiabetic model (Fig. 4D–F; Fig. S1B). Fluorescence intensity of Rbfox1 in rat VSMCs or tissues also exhibited a downregulation in HFD/STZ rats in comparison to control rats (Fig. S5). Unexpectedly, the arteries from the patients with diabetes showed increased Rbfox1 expression in comparison to those from nondiabetic patients (Fig. S6B, C). We consider this might be due to compensatory effects, though the underlying mechanisms are unknown. Nevertheless, our data indicate that decrease in Rbfox1 might play a crucial role in the regulation of *Cacna1c* AS events in the diabetic arteries.

### GS induces decreased Rbfox1 expression and aberrant Ca_V_1.2 AS in VSMCs and MAs

**Fig. 5:**
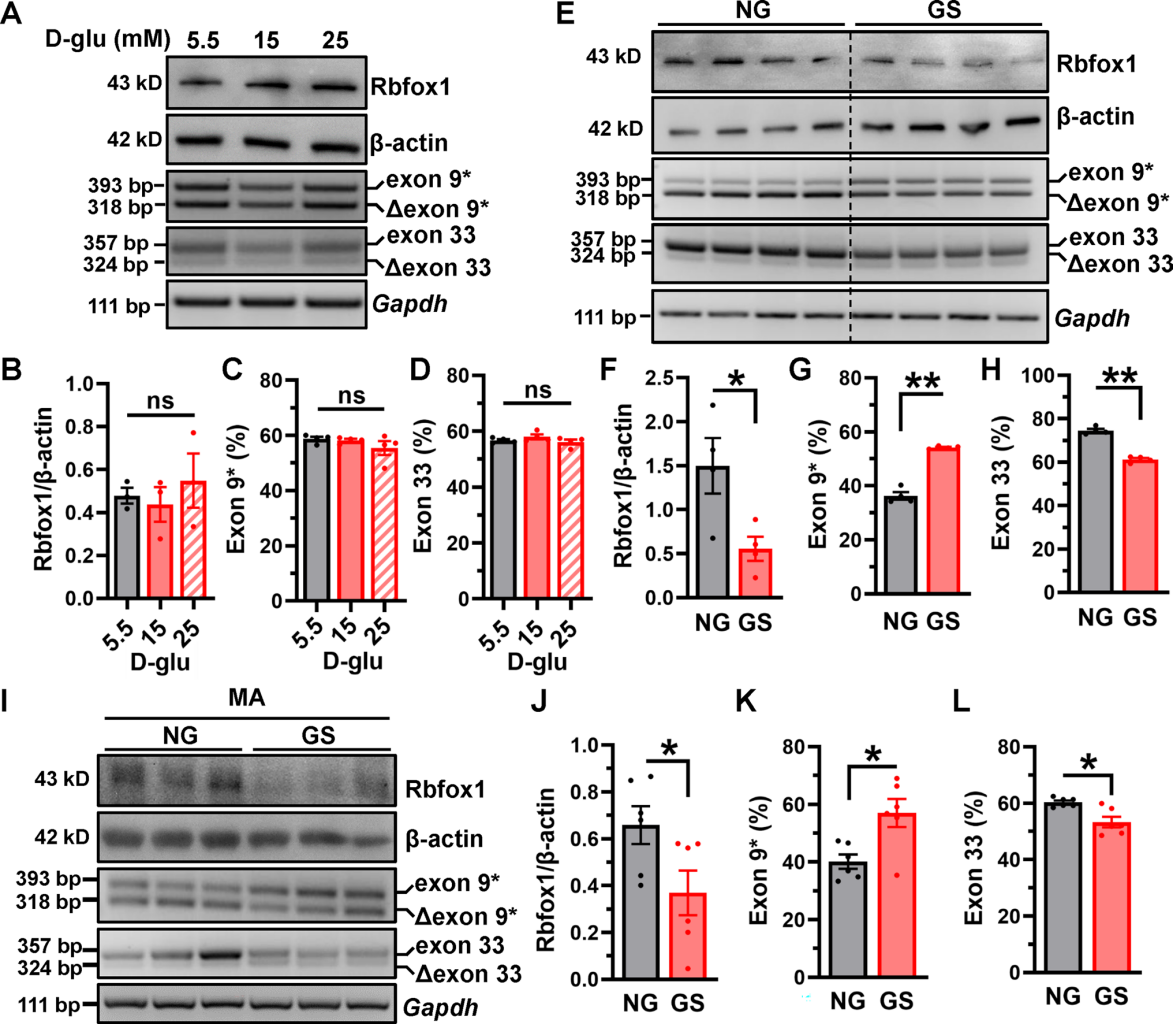
GS, not glucose, decreases Rbfox1 expression in VSMCs and MAs. **A** Isolated VSMCs were treated with 5.5, 15 or 25 mmol/L d-glucose for 48 h and Western blotting were used to detect the expression of Rbfox1. β-actin was detected as an internal control. **B** The expression level of Rbfox1 was checked in VSMCs after the application of d-glucose. The proportion of Ca_V_1.2 with alternative exon 9* (**C**) and exon 33 (**D**) were detected by RT-PCR. ns indicates no significant differences. 1-way ANOVA followed by a Tukey’s post hoc test. **E** VSMCs were treated with 20% NG or GS for 48 h and Western blotting was performed to detect the expression of Rbfox1. The proportion of Ca_V_1.2 with alternative exon 9* or exon 33 were detected by RT-PCR. **F** The relative Rbfox1 expression was normalized in VSMCs after treatments. The values for percent exon 9* (**G**) or exon 33 inclusion (**H**) of Ca_V_1.2 channels are presented as bar charts. The ratios between the upper band and the sum of the upper and lower bands present the proportions of Ca_V_1.2 channel with exon 9* or exon 33. **I** MAs were treated with 20% NG or GS for 48 h and Western blotting was performed to detect the expression of Rbfox1. The proportion of Ca_V_1.2 with exon 9* or exon 33 were detected by RT-PCR. **J** The relative Rbfox1 expression was normalized to β-actin after treatments. The values for percent exon 9* (**K**) or exon 33 inclusion (**L**) of Ca_V_1.2 channels were presented as bar charts. * *P* < 0.05, ** *P* < 0.01 vs NG-treated VSMCs and MAs, unpaired *t* test with or without Welch’s correction

Since Rbfox1 expression was also reduced in the hypertensive arteries [33], we questioned whether the decreased Rbfox1 in diabetic arteries was due to diabetic hyperglycemia or diabetes-associated hypertension. To evaluate this, we used a cell-based model, treating isolated VSMCs from rat MAs with HG (15 or 25 mmol/L) (Fig. 5A). Unexpectedly, the application of HG did not alter the expression level of Rbfox1 in isolated VSMCs (Fig. 5B), nor the expression of Ca_V_1.2 alternative exon 9* (Fig. 5C) and exon 33 (Fig. 5D). This suggest that HG may not directly affect Ca_V_1.2 AS events of VSMCs in diabetic arteries.

Abnormal vascular functions are known to be driven by AGEs under diabetic hyperglycemic conditions [5]. To explore the role of AGEs in Rbfox1 expression and Ca_V_1.2 AS events, we used GS to mimic AGEs-related modification in diabetes. Isolated VSMCs from rat MAs were treated with 20% GS or NG for 48 h. The expression of Rbfox1 was found to be notably decreased after GS application when compared with NG application (Fig. 5E, F). Significantly, GS treatment increased Ca_V_1.2_E9*_ channels (Fig. 5G) but decreased Ca_V_1.2_E33_ channels in VSMCs (Fig. 5H). However, mannitol didn’t affect the expression levels of Rbfox1 in VSMCs, eliminating the possible interference of osmotic pressure (Fig. S7A, B). GS decreased Rbfox1 expression (Fig. S7C, D) and regulated Ca_V_1.2 alternative exon 9* in a concentration-dependent manner, but it did not regulate Ca_V_1.2 alternative exon 33 (Fig. S7E–G). Ca_V_1.2 exons 9* and 33 AS events did not change in VSMCs when cultured for 24 or 48 h compared to freshly isolated cells (0 h) (Fig. S8). This indicates the cell culture didn’t alter Ca_V_1.2 AS events. Furthermore, the application of GS in the MAs also induced a reduction in Rbfox1, and increased Ca_V_1.2 exon 9*, but decreased exon 33 (Fig. 5I–L). These results were consistent with the findings that diabetic arteries exhibit decreased Rbfox1 expression and aberrant splicing of the Ca_V_1.2 channel, and imply that GS, not glucose, induces the aberrant splicing of the Ca_V_1.2 channel, which may be related to enhanced channel functions.

In order to eliminate the interference of Ca_V_1.2 phosphorylation induced by GS, we examined the phosphorylation level of Ca_V_1.2 at S1928 in VSMCs treated with GS. Here, we found GS does not increase the expression level of Ca_V_1.2 S1928. However, co-treatment with PKA inhibitor H-89 significantly decreased its expression level (Fig. S9), indicating that Rbfox1-mediated exon splicing is a long-acting mechanism. As the chronic effects of diabetes in enhanced arterial contraction via Ca_V_1.2 had been demonstrated to require phosphorylation at S1928 in a model of STZ-treated mice [14], our observation might be an alternative, compatible mechanism involved in the increased arterial contractility.

Additionally, MGO, a highly reactive dicarbonyl compound that forms AGEs [39], was used to treat VSMCs for 48 h. After that, the expression of Rbfox1 also showed a significant decrease (Fig. S10A, B), while both Ca_V_1.2_E9*_ and Ca_V_1.2_E33_ channels exhibited an increase when compared with vehicle-treated cells (Fig. S10C, D). These observations further confirmed that the aberrant expressions of Rbfox1 and Ca_V_1.2 AS are indeed due to AGEs.

### Overexpression of Rbfox1 reverses aberrant splicing of Ca_V_1.2 channels in GS-treated VSMCs

**Fig. 6:**
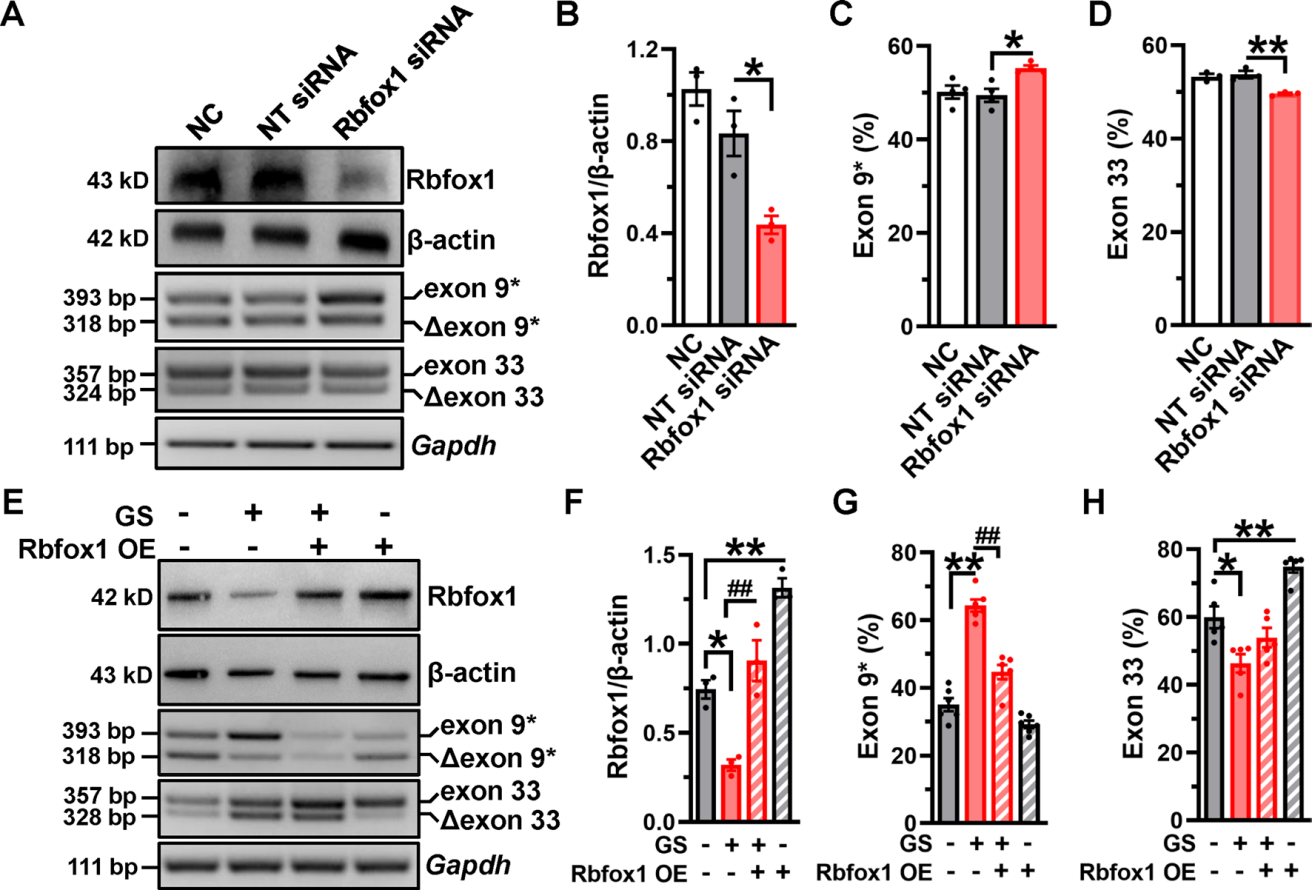
Rbfox1 dynamically regulates alternative exons 9* and 33 of Ca_V_1.2 channel in VSMCs. **A** NT or Rbfox1 siRNAs were transfected into isolated VSMCs. After 48 h of culture, the Rbfox1 protein and alternative exon 9* or exon 33 of Cav1.2 were detected by Western blotting and RT-PCR, respectively. **B** The relative Rbfox1 expression is presented in VSMCs after different treatments. The values for percent exon 9* (**C**) or exon 33 inclusion (**D**) are presented as bar charts. * *P* < 0.05, ** *P* < 0.01 vs NT siRNA-treated VSMCs, 1-way ANOVA followed by a Tukey’s post hoc test. **E** VSMCs treated with GS were transfected with Rbfox1 expression plasmids for 48 h, then the Rbfox1 protein and alternative exon 9* or exon 33 of Ca_V_1.2 were detected by Western blotting and RT-PCR, respectively. **F** Rbfox1 expression level was normalized to β-actin expression in VSMCs after GS treatment. The percentages of exon 9* (**G**) or exon 33 inclusion (**H**) are shown as bar charts. The ratios between the upper band and the sum of the upper and lower bands present the proportions of Ca_V_1.2 channel with exon 9* or exon 33. * *P* < 0.05, ** *P* < 0.01 vs control, ^##^ *P* < 0.01 vs GS-treated VSMCs, 1-way ANOVA followed by a Tukey’s post hoc test. *n* represents the number of cells

We then examined the direct effects of Rbfox1 on Ca_V_1.2 AS events. An siRNA approach was used to specifically knockdown the endogenous expression of Rbfox1 in VSMCs (Fig. 6A, B). RT-PCR results showed that the proportion of Ca_V_1.2_E9*_ channel increased (Fig. 6C), but Ca_V_1.2_E33_ channel decreased (Fig. 6D). Moreover, Rbfox1 siRNA RP in MAs significantly increased Ca_V_1.2_E9*_ channels, but decreased Ca_V_1.2_E33_ channels (Fig. S11). These results indicated that Rbfox1 could directly regulate AS events of the Ca_V_1.2 channel in VSMCs and in arteries. Furthermore, transfection with Rbfox1 expression plasmids staved off GS-induced Rbfox1 downregulation (Fig. 6E, F), and abolished GS-induced Ca_V_1.2_E9*_ increase (Fig. 6G) and Ca_V_1.2_E33_ decrease (Fig. 6H), partly reversing the aberrant expressions of exons 9* and 33 induced by GS application in VSMCs. Altogether, these results revealed that the aberrant splicing of Ca_V_1.2 in diabetic arteries is indeed attributed to the decreased Rbfox1.

### GS hyperpolarizes Ca_V_1.2 *I*–*V* curve in VSMCs

**Fig. 7:**
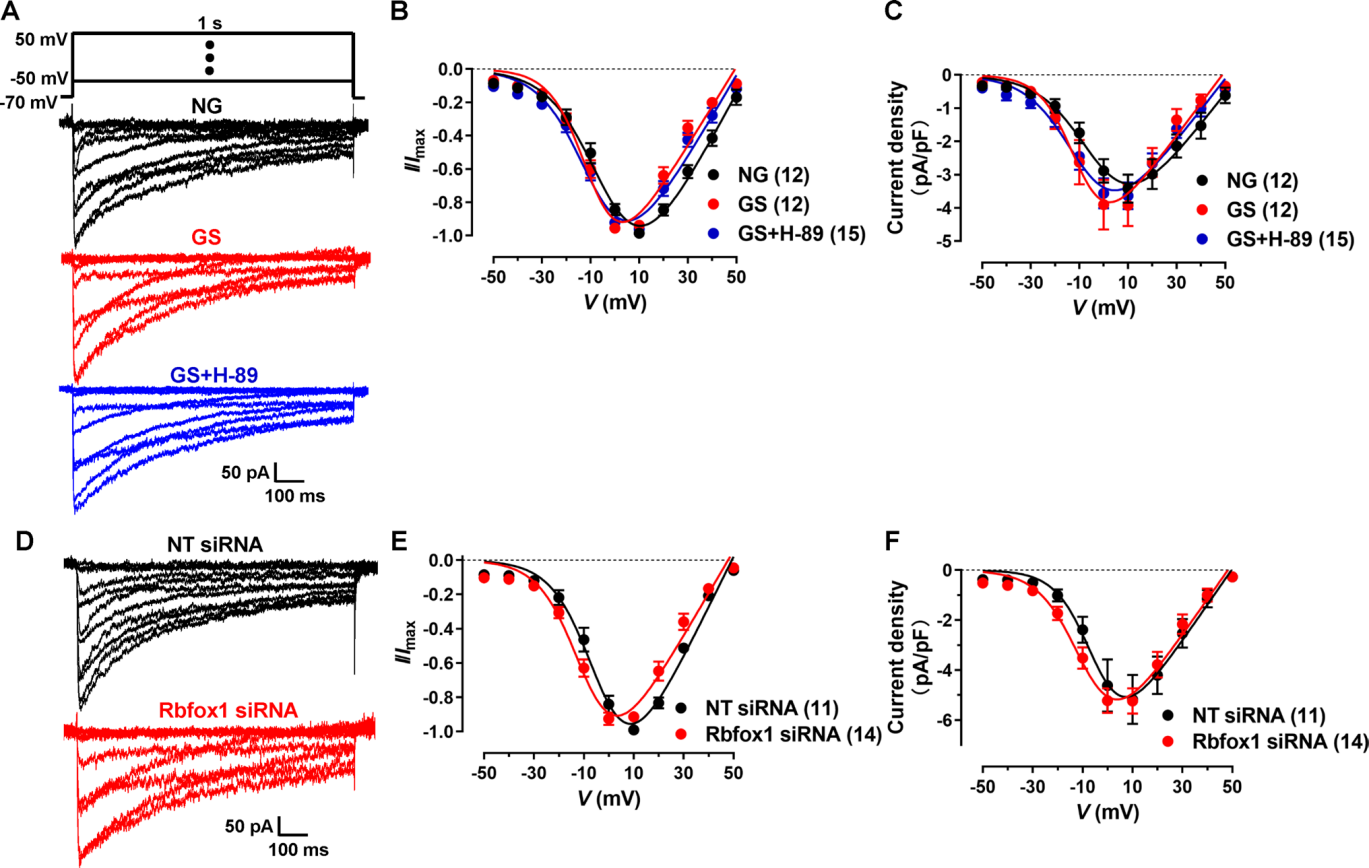
Treatment with GS and knockdown of Rbfox1 lead to hyperpolarization of Ca_V_1.2 *I*–*V* curve in VSMCs. **A** Ca_V_1.2 channel currents were recorded under the testing potentials, increasing from − 50 to 50 mV (10-mV increase each step, *I*–*V* protocol) in aortic VSMCs when using 10 mmol/L Ba^2+^ as charger carrier. Representative traces of Ca_V_1.2 currents were presented after NG or GS treatment for 48 h. Additionally, 50 μmol/L PKA inhibitor H-89 was administrated 4 h before the recording in GS-treated VSMCs. **B** Plots of Ca_V_1.2 *I*–*V* curve of VSMCs were analyzed after treating with NG, GS or GS plus H-89. **C** Current densities of Ca_V_1.2 channel were calculated by dividing currents by cell capacitance (*C*_m_) of the VSMCs after applying with NG, GS or GS plus H-89. **D** Representative traces of Ca_V_1.2 currents under *I*–*V* protocol were shown in NT or Rbfox1 siRNA-transfected VSMCs. **E** Plots of Ca_V_1.2 *I*–*V* curve of VSMCs were analyzed and fitted with the equation. **F** Current densities of Ca_V_1.2 channel were calculated by dividing by cell capacitance after applying with NT or Rbfox1 siRNAs in VSMCs. ns indicates no significant differences. *n* represents the number of cells

To determinate whether AGEs affect the functions of vascular Ca_V_1.2 channels, isolated VSMCs from rat aortas were treated with 20% GS or NG for 24–48 h. Then, Ca_V_1.2 channel currents were recorded upon increasing step voltages using a whole-cell patch clamp. As indicated in Fig. 7A and B, GS-treated VSMCs showed an obviously left-shifted *I*–*V* relationship curve compared to NG-treated cells (Table S6). However, their current densities of Ca_V_1.2 channels showed no significant differences (Fig. 7C). As the phosphorylation of Ca_V_1.2 α_1C_ at S1928 caused by PKA could increase Ca_V_1.2 currents of VSMCs in diabetic arteries [12], we used PKA specific inhibitor H-89 to block the PKA-mediated phosphorylation of Ca_V_1.2 channels. The data showed that H-89 application didn’t affect Rbfox1 expression and Ca_V_1.2 alternative exon 9* and exon 33 (Fig. S12), and couldn’t restore the left-shifted Ca_V_1.2 *I*–*V* relationship curve in GS-treated VSMCs (Fig. 7A, B), indicating PKA signaling doesn’t mediate the Ca_V_1.2 channel kinetics changes in diabetic arteries. GS application increased the cell capacitance of VSMCs (Fig. S3B). Similar results were observed in the MGO-treated VSMCs as shown in Fig. S10E–G and Table S7, but the cell capacitance remained unchanged (Fig. S3C). These observations indicated that AGEs could hyperpolarize Ca_V_1.2 *I*–*V* curve in VSMCs, which is not associated with PKA-mediated protein phosphorylation.

To explore the electrophysiological relevance of Rbfox1 in Ca_V_1.2 channel, we used siRNAs to knockdown Rbfox1 in isolated VSMCs. Rbfox1 knockdown obviously left-shifted the *I*–*V* curve in comparison to NT siRNA-treated VSMCs (Fig. 7D, E; Table S8). However, the cell capacitance (Fig. S3D) and current densities (Fig. 7F) of Ca_V_1.2 channels after Rbfox1 knockdown remained unchanged, indicating that decreased Rbfox1 could directly hyperpolarize Ca_V_1.2 channel *I*–*V* kinetics without affecting its current size in VSMCs. Thus, it is reasonable to conclude that the GS-induced hyperpolarized Ca_V_1.2 *I*–*V* curve is due to decreased Rbfox1 in VSMCs, and the hyperpolarized *I*–*V* and activation curve of Ca_V_1.2 channels in VSMCs from rat diabetic arteries is attributed to decreased Rbfox1 expression.

### GS increases K^+^-induced [Ca^2+^]_*i*_ and vascular constriction

**Fig. 8:**
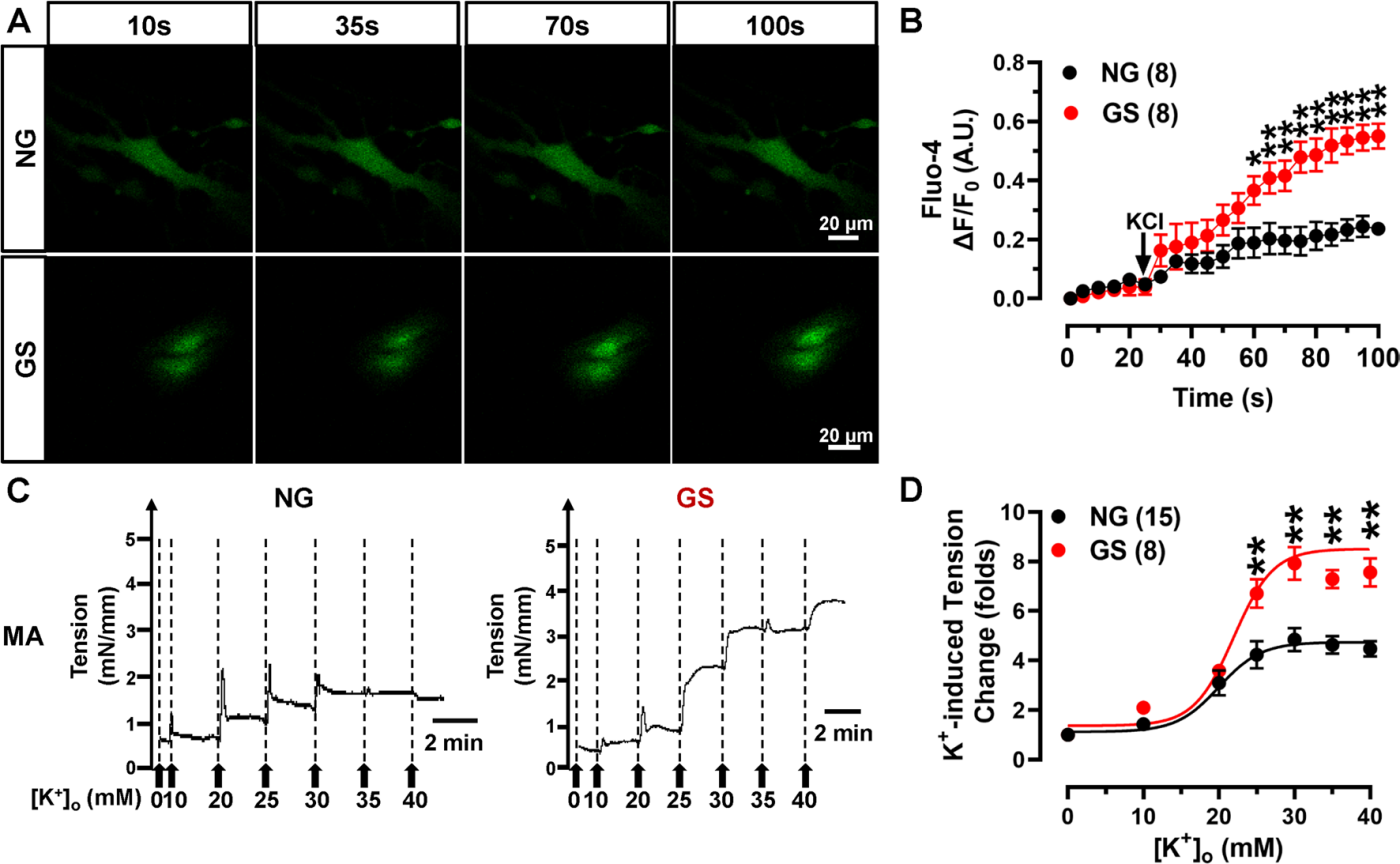
Application with GS elevates K^+^-induced [Ca^2+^]_*i*_ of VSMCs and enhances vasoconstriction. **A** VSMCs were treated with 20% NG or GS for 48 h, and [Ca^2+^]_*i*_ triggered by 60 mmol/L KCl was measured using Ca^2+^ fluorescence indicator Fluo-4 AM. The fluorescent intensity was monitored using a time series scanning mode under a confocal microscope. **B** Δ[Ca^2+^]_*i*_ was presented as Δ*F*/*F*_0_ and shown as a line diagram, * *P* < 0.05, ** *P* < 0.01 vs NG-treated VSMCs, 2-way ANOVA followed by Sidak’s multiple comparisons. *n* is the number of cells from at least 3 rats. **C** Isolated mesenteric arteries were cultured with 20% NG or GS for 48 then the force tension induced by increasing extracellular K^+^ concentration was recorded using vascular myograph. **D** Plots of concentration-tension relationship were represented and fitted with the Boltzmann equation for NG or GS-treated arteries. ** *P* < 0.01 vs NG-treated arteries, 2-way ANOVA followed by Sidak’s multiple comparisons. *n* represents the number of rats

Ca^2+^ influx via the Ca_V_1.2 calcium channel triggers the vasoconstriction and maintain the myogenic tone. Hyperglycemia or GS treatment can induce the hyperpolarization of vascular Ca_V_1.2 *I*–*V* kinetics, making the channels open more easily from resting membrane potential, which might increase the [Ca^2+^]_*i*_ in VSMCs. To test this possibility, we incubated VSMCs with Fluo-4 AM, and applied 60 mmol/L KCl to trigger the elevation of [Ca^2+^]_*i*_ (Fig. 8A). Co-incubation with 20% GS could increase [Ca^2+^]_*i*_ in VSMCs. Though the current size of Ca_V_1.2 channel was decreased in VSMCs from HFD/STZ-treated rats (Fig. 2C), the present data supported the result that GS could hyperpolarize Ca_V_1.2 *I*–*V* curve, which could also increase the [Ca^2+^]_*i*_ in VSMCs (Fig. 8B). To confirm that the [Ca^2+^]_*i*_ increase in GS-treated vessels indeed represents a larger Ca^2+^ influx and is not due to differences in *F*_0_, we pretreated the isolated VSMCs with nifedipine, the results showed that application with 10^−6^ mol/L nifedipine could significantly block the GS-induced increase in [Ca^2+^]_*i*_, indicating the larger [Ca^2+^]_*i*_ increase in GS-treated VSMCs is indeed trigged by Ca_V_1.2-mediated Ca^2+^ influx (Fig. S13).

We next examined the effects of GS application on the vasoconstriction of small arteries. MAs were isolated from the rats and cultured with 20% GS or NG for 48 h. Subsequently, increasing concentrations of KCl were applied to induce vasoconstriction (Fig. 8C). In comparison to NG-treated arteries, GS application significantly increased K^+^-induced vasoconstriction in MAs (Fig. 8D). However, applying the PKA inhibitor H-89 didn’t change the vascular tension of GS-treated MAs (Fig. S14), indicating that PKA signaling doesn’t mediate GS-induced vasoconstriction. Combined with the finding that GS did not increase the level of Ca_V_1.2^S1928^ phosphorylation, we confirmed that Rbfox1-mediated Ca_V_1.2 AS is involved in the long-acting mechanisms regulating vasoconstriction. Moreover, we introduced Rbfox1 expression plasmids into isolated MAs from GK rats, and found that the enhanced vasoconstriction in diabetic rats was restored (Fig. S1C, D). Our previous finding showed that the knockdown of Rbfox1 increased K^+^-induced vasoconstriction [33]. Together, these results indicated that GS application can directly enhance the vascular constriction, which is thought to be mediated by aberrant splicing of Ca_V_1.2 channels induced by decreased Rbfox1.

## Discussion

**Fig. 9:**
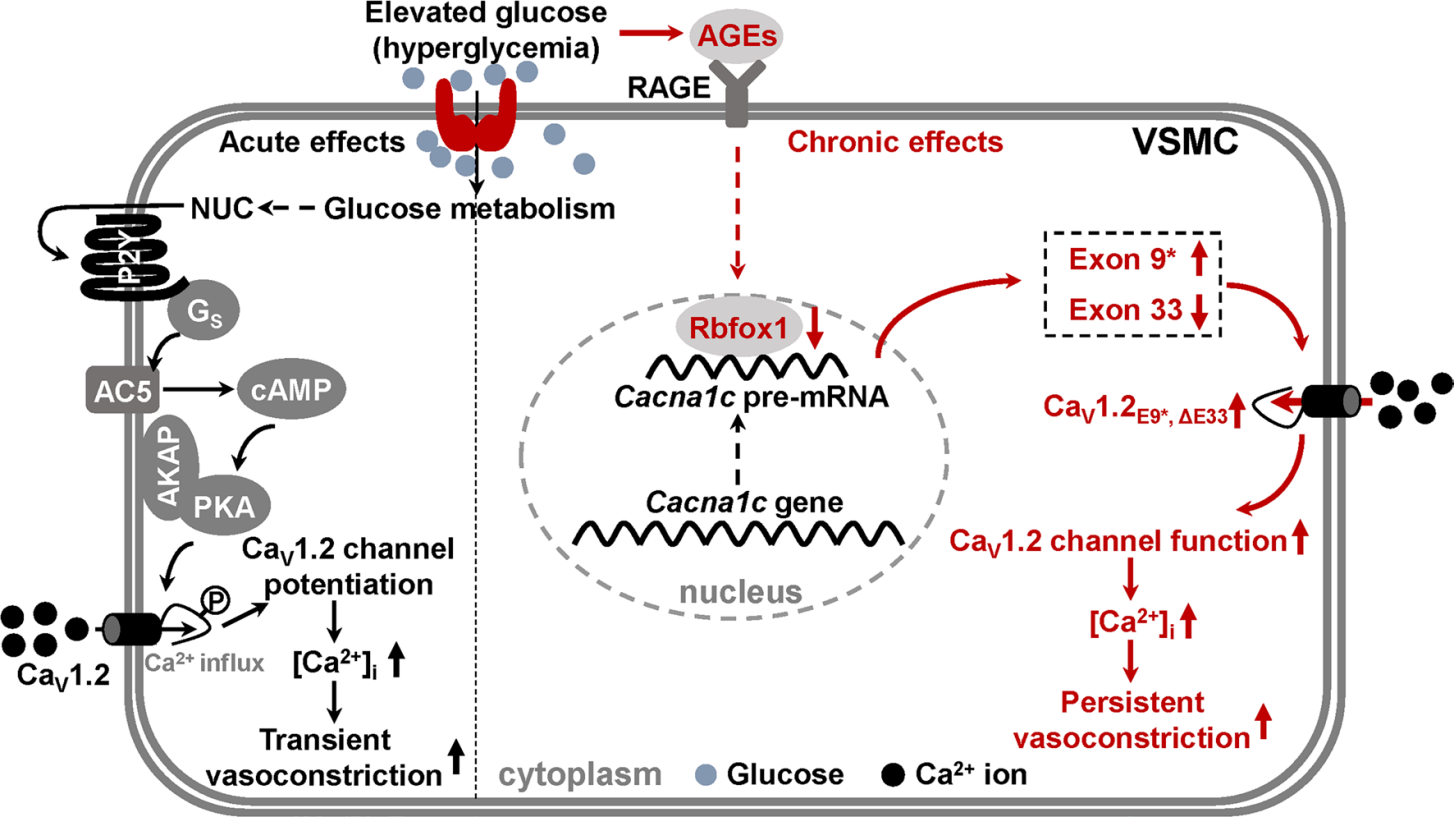
Illustration of main findings in the present study. Schematic diagram illustrates the acute and long-term mechanisms that regulate vascular Ca_V_1.2 functions and vasoconstriction under diabetic hyperglycemia. For the long-lasting mechanism, AGEs significantly decrease Rbfox1 expression, and enhance the function of vascular Ca_V_1.2 channel by dynamically regulating alternative exons 9* and 33, thereby inducing persistent vasoconstriction during diabetic hyperglycemia. NUC, nucleotide; RAGE, receptor for advanced glycation end-product; VSMC, vascular smooth muscle cell

The Ca_V_1.2 channel is indispensable for the excitation–contraction coupling of vascular smooth muscle [40], and its abnormal functions are tightly associated with increased vasotone [41, 42]. The Ca_V_1.2 channel is regulated by many post-transcriptional modulations, including AS [43]. We previously discovered aberrant AS events of the Ca_V_1.2 channel under cardiovascular pathological conditions, such as cardiac hypertrophy [26] and hypertension [24, 28, 33]. In this study, we present, for the first time to our knowledge, that AS of vascular Ca_V_1.2 channel is dysregulated under hyperglycemic condition. This dysregulation facilitates channel function by hyperpolarizing the Ca_V_1.2 channel activation kinetics, leading to persistently enhanced vasoconstriction of diabetic arteries (Fig. 9). Since most of our cell-based experiments were performed in the cultured cells, and AS of vascular Ca_V_1.2 channel may play an important role in the proliferation of smooth muscle cells triggered by calcium influx. Therefore, this AS modulation in Ca_V_1.2 channels might also present in proliferation, not only in contractility of vascular smooth muscle, which needs further investigations. Nevertheless, our findings provide a new perspective on the precise regulatory mechanism of vascular Ca_V_1.2 channel activity in response to diabetic hyperglycemia.

Dysfunctions of smooth muscle are recognized to play significant roles in diabetic vascular complication [44]. In vivo measurements in diabetic animal models have shown increased vasotone [13]. Here, we observed that isolated arteries from HFD/STZ-treated diabetic rats and type 2 diabetic GK rats exhibited persistent constriction in response to K^+^, reinforcing the correlation between enhanced vasoconstriction and vascular smooth muscle in diabetes. Moreover, GS treatment appeared to induce elevated K^+^-triggered vasoconstriction, which is believed to be mediated by the long-term regulatory mechanism in the arteries.

The abnormal expression and functioning of the Ca_V_1.2 channel in arteries is associated with enhanced vasotone, which is a crucial factor in the development of hypertension [28, 33]. Under the conditions of diabetic hyperglycemia, the Ca_V_1.2 channel currents are known to increase in response to elevated extracellular glucose. This is dependent on the phosphorylation of Ca_V_1.2^S1928^, which is mediated by AKAP5/P2Y_11_/AC5/PKA signaling, thus leading to an increase in vasotone [12, 13, 45, 46]. Considering that phosphorylation process of threonine and serine amino acids required up to dozens of minutes [47], increased Ca_V_1.2 channel function responded to hyperglycemia might be an acute effect. In this work, we found that long-term incubation with GS did not increase the level of Ca_V_1.2^S1928^ phosphorylation, indicating that facilitated function of vascular Ca_V_1.2 channel is mainly because of Rbfox1-mediated alternative splicing, not PKA-mediated S1928 phosphorylation. Also, we found that the *I*–*V* and steady-state activation curve of Ca_V_1.2 channels shifted towards the left (hyperpolarized) in HFD/STZ-treated rat VSMCs. Moreover, GS treatment directly hyperpolarized the Ca_V_1.2 *I*–*V* curve in isolated VSMCs, which was independent of PKA signaling, indicating a long-term modulation mechanism in regulating the function of Ca_V_1.2 channel under diabetic hyperglycemia. Therefore, we proposed that the dual mechanisms (acute and long-term) for the regulation of vascular Ca_V_1.2 functions might finely adjust the acute and persistent vasoconstriction of small arteries in response to hyperglycemia, respectively.

Under diabetic hyperglycemia, the vascular Ca_V_1.2 channel shows enhanced function by hyperpolarizing the channel activation kinetics. The Ca_V_1.2 alternative exon 9* increases aberrantly, while exon 33 decreases, generating the upregulation of a specific Ca_V_1.2 isoform, the Ca_V_1.2_E9*_,_ΔE33_ channel. We previously found that the electrophysiological properties of the Ca_V_1.2_E9*_,_ΔE33_ isoform, when heterologously expressed in human embryonic kidney-293 cells, show hyperpolarized *I*–*V* and steady-state activation curves compared to the Ca_V_1.2_ΔE9*_,_E33_ isoform [28]. This hyperpolarized Ca_V_1.2 *I*–*V* and steady-state activation leads to the channel opening much more easily from the resting membrane potential. Therefore, we conclude that the facilitated function of the vascular Ca_V_1.2 channel is truly attributed to the upregulation of Ca_V_1.2_E9*_,_ΔE33_ isoform under diabetic hyperglycemia. As HG could depolarize the membrane potential of VSMCs [14], this may further augment the functions of vascular Ca_V_1.2 channels due to the upregulated Ca_V_1.2_E9*_,_ΔE33_ isoforms in diabetes. The electrophysiological properties of Ca_V_1.2 channel differentiate between diabetic rats and VSMCs in vitro. This discrepancies in Ca_V_1.2 channel activity could be associated with the specific animal model and concurrent metabolic abnormalities, such as hypercholesterolemia and hyperinsulinemia, which could affect channel functions [48]. GS and Rbfox1 knockdown did not induce any change in current density, thus we speculated that reduced current densities in smooth muscle cells from HFD/STZ-induced diabetic rats may be attributed to other metabolic abnormalities, such as hypercholesterolemia or hyperinsulinemia. On the other hand, the 48-h serum culture may also have resulted in a phenotypic switch towards a non-contractile/synthetic phenotype. This shift could potentially account for the observed lack of changes in Ca_V_1.2 channel current.

Splicing factors produce different actions by binding to different positions on pre-mRNA (position-dependent) [49]. Several UGCAUG elements surround exon 9* and exon 33 of *Cacna1c* gene. Rbfox proteins prefer binding upstream of exon 9* to repress its expression, but downstream of exon 33 to enhance its expression [32]. Here, we found that alternative exon 9* is increased, but exon 33 is decreased. This is accompanied by reduced Rbfox1 expression in the arteries of HFD/STZ-induced and GK rats. Moreover, Rbfox1 knockdown in isolated VSMCs directly induces the upregulation of exon 9* and downregulation of exon 33. Although we cannot exclude the possibility that Rbfox1 mediates the AS events of other target genes [50–52] in diabetic arteries, our observations are sufficient to conclude that decreased Rbfox1 in VSMCs indeed induces aberrant expression of the vascular Ca_V_1.2_E9*_,_ΔE33_ channel under diabetic hyperglycemia, which in turn enhances vasoconstriction. Notably, several variants of *RBFOX1* gene are linked to lower blood pressure in humans [53, 54]. Whether these variants influence Rbfox1-mediated Ca_V_1.2 AS in diabetic arteries needs further studies.

The different patterns of increased Ca_V_1.2 exon 33 induced by MGO and decreased exon 33 induced by diabetes and GS suggest that potential factors may modulate the expression of Ca_V_1.2 exon 33 compare with exon 9*. Although we confirmed the important role of Rbfox1 in regulating Ca_V_1.2 functions and vasoconstriction under diabetic hyperglycemia in the present study, we also found another splicing factor, Rbfox2, dynamically regulating Ca_V_1.2 exons 9* and 33 in VSMCs [28]. Besides Rbfox proteins, other splicing factors such as RNA-binding protein motif 20 (RBM20) has also been reported to regulate Ca_V_1.2 alternative exon 8, exon 9*, exon 22 and exon 33, but the magnitude of this regulation is weak [55]. It is not clear whether other splicing factors specifically control the expression of Ca_V_1.2 exon 33, but this requires further clarification.

We initially hypothesized that HG in diabetic condition may mediate the decreased expression of Rbfox1 in the arteries from diabetic rats. Contrary to our expectations, HG didn’t directly affect Rbfox1 expression and the Ca_V_1.2 splicing pattern in VSMCs. AGEs are produced during chronic hyperglycemic condition, causing long-lasting damage to vascular complications through metabolic memory [15]. Treatment with GS, an AGEs mimic, significantly decreased the expression of Rbfox1 and increased the presence of the Ca_V_1.2_E9*_,_ΔE33_ isoform in isolated VSMCs. Functionally, the application of GS shifted the Ca_V_1.2 *I*–*V* curve towards hyperpolarization in isolated VSMCs, enhancing channel function and increasing K^+^-induced vasoconstriction. These observations confirm that AGEs can induce a reduction in Rbfox1 protein through metabolic memory, resulting in aberrant splicing and function of the vascular Ca_V_1.2 channel under diabetic hyperglycemia.

## Conclusions

In summary, our research shows that AGEs can significantly reduce Rbfox1 expression and enhance the function of vascular Ca_V_1.2 channel by dynamically regulating alternative exons 9* and 33, thereby increasing vasoconstriction in the arteries of diabetic rat. Therefore, targeting Rbfox1 to correct the aberrant splicing of the Ca_V_1.2 channel in vascular smooth muscle may provide a promising approach for the management of diabetic vascular complications.

### Supplementary Information

Below is the link to the electronic supplementary material.

Supplementary file1 (PDF 2230 KB)

## Data Availability

The data sets generated and/or analyzed during this study are available from the corresponding author upon reasonable request.
