# Supplementary material for: Aberrant splicing of CaV1.2 calcium channel induced by decreased Rbfox1 enhances arterial constriction during diabetic hyperglycemia
Source: Cell Mol Life Sci. 2024 Apr 4;81(1):164. doi: 10.1007/s00018-024-05198-z (PMC10995029; doi:10.1007/s00018-024-05198-z)
Supplement: Supplementary file 1 — Supplementary file1 (PDF 2230 KB) [file 18_2024_5198_MOESM1_ESM.pdf]

Supplementary Information

Aberrant splicing of $Ca_V1.2$ calcium channel induced by decreased Rbfox1 enhances arterial constriction during diabetic hyperglycemia

Wei Hou[1,2,3], Shumin Yin[1,2], Pengpeng Li[1,2], Ludan Zhang[1,2], Tiange Chen[1,2], Dongxia Qin[1,2], Atta Ul Mustafa[1,2], Caijie Liu[1,2], Miaomiao Song[1,2], Cheng Qiu[4], Xiaoqing Xiong[1,2,3*], Juejin Wang[1,2,3*]

[1]Key Laboratory of Targeted Intervention of Cardiovascular Disease, Collaborative Innovation Center for Cardiovascular Disease Translational Medicine, Nanjing Medical University, Nanjing, Jiangsu, China;

[2]Department of Physiology, Nanjing Medical University, Nanjing, Jiangsu, China;

[3]The Affiliated Taizhou People's Hospital of Nanjing Medical University, Taizhou School of Clinical Medicine, Nanjing Medical University, Taizhou, Jiangsu, China;

[4]Nanjing Comprehensive Stroke Center, Affiliated Nanjing Brain Hospital, Nanjing Medical University, Nanjing, Jiangsu, China.

*Correspondence:

Juejin Wang, Department of Physiology, Nanjing Medical University, 101 Longmian Ave, Nanjing 211166, Jiangsu, China. Email: juejinwang@njmu.edu.cn

or Xiaoqing Xiong, Department of Physiology, Nanjing Medical University, 101 Longmian Ave, Nanjing 211166, Jiangsu, China. Email: xqxiong@njmu.edu.cn

Table S1. Clinical characteristics of the patients

Sample	Sex	Age (y)	Relevant Medical History	Glucose level (mmol/L)	Pre-op medications
**1**	F	58	Hypertension, **Diabetes**	6.79	/
**2**	F	64	Hypertension, **Diabetes**	6.70	Metformin Amlodipine besylate
**3**	F	66	Cerebral infarction, **Diabetes** Cholecystectomy, Hysterectomy, Moyamoya disease	10.54	/
**4**	F	72	Hypertension, **Diabetes** left middle cerebral occlusion	7.12	Nifedipine, Metformin, Acarbose
**5**	M	53	Moyamoya disease, **Diabetes**	7.3	Insulin
**6**	M	56	Moyamoya disease	4.34	/
**7**	F	47	Moyamoya disease	5.55	/
**8**	F	42	Moyamoya disease	3.84	/
**9**	M	66	Moyamoya disease, Cerebral infarction, Hypertension	5.01	/
**10**	M	54	Moyamoya disease, Pulmonary emphysema, Duodenal ulcer	4.72	/

Table S2. Sequences of siRNAs targeting with rat Rbfox1 mRNA

siRNAs	Sense/antisense	Sequences
#1 siRNA	Sense<br>Antisense	5′ CGAGGUUAAUAAUGCGACAdTdT 3′<br>3′ dTdTGCUCCAAUUAUUACGCUGU 5′
#2 siRNA	Sense<br>Antisense	5′ GGAUCCAGACCUCCGACAAdTdT 3′<br>3′ dTdTCCUAGGUCUGGAGGCUGUU 5′
#3 siRNA	Sense<br>Antisense	5′ CGGUGUUGUUUACCAGGAUdTdT 3′<br>3′ dTdTGCCACAACAAAUGGUCCUA 5′

Table S3. Primer sequences for PCR

Target genes	Oligonucleotide primers (5'-3')	Product length (bp)
Rat *Cacna1c* exon 8	FP: TCAATGATGCCGTAGGAAG<br>RP: CGCTAAGAACACCGAGAA	102
Rat *Cacna1c* exon 8a	FP: ACGCTATGGGCTATGAGT<br>RP: GGTGATCCAGTCCAGGTA	206
Rat *Cacna1c* exon 9*/Δ9*	FP: TTCCAAAGAGAGGGAGAAAGC<br>RP: CTGCAGAATCTATTCCACCGG	393 (E9*)<br>318 (ΔE9*)
Rat *Cacna1c* exon 33/Δ33	FP: GCCTCTTCACGGTGGAG<br>RP: TCCCAATCACTGCATAGATAA	357 (E33)<br>324 (ΔE33)
Human *Cacna1c* exon 9*/Δ9*	FP: TTCCAAAGAGAGGGAGAAGGC<br>RP: CTGCAGAACCGATTCCACCGG	393 (E9*)<br>318 (ΔE9*)
Human *Cacna1c* exon 33/Δ33	FP: GCCTGTTCAAAATCGCC<br>RP: TCCCGATCACCGCGTAGATGA	402 (E33)<br>369 (ΔE33)
Rat *Gapdh*	FP: CAGGGCTGCCTTCTCTTGTG<br>RP: AACTTGCCGTGGGTAGAGTC	111

Table S4. The information of key antibodies used in this study

	**Western blotting**		**Immunofluorescence**	
**Target protein**	**Rbfox1**	**β-actin**	**Rbfox1**	**α-SMA**
**Vendor**	ThermoFisher (MA533104)	Abways Technology (AB0035)	Santa Cruz Biotechnology (sc-135476)	Novus Biologicals (NBP2-33006)
**Species reactivity**	Human, Mouse, Rat	Human, Mouse, Rat	Mouse, Rat	Mouse, Rat
**Host/Subtype**	Mouse IgG1	Rabbit IgG	Rabbit IgG	Mouse IgG
**Classification**	Monoclonal	Monoclonal	Monoclonal	Monoclonal
**Purification**	Protein G	Affinity-chromatography	Protein G	Protein A
**Antigen**	Recombinant protein taken from the N-terminus of human FOX1 expressed in and purified from E. Coli	A synthesized peptide derived from human beta Actin	Amino acids 38-118 mapping near the N-terminus of Fox-1 of human	N-Terminal decapeptide of Alpha smooth muscle isoform of actin

Table S5. Summary of Cav1.2 channel electrophysiological characteristics of isolated VSMCs from control or HFD/STZ-treated rats MAs

	$V_{0.5}$ (mV)	E_{rev} (mV)	k	n
Ctrl	-4.74±2.19	54.85±1.57	-12.35±0.92	9
HFD/STZ	-12.13±1.68*	57.08±1.77	-11.19±0.85	9
	$V_{0.5,act}$ (mV)	n	$V_{0.5,inact}$ (mV)	n
Ctrl	18.88±4.42	11	-16.50±1.94	7
HFD/STZ	5.94±3.94*	10	-20.25±2.59	5

$V_{0.5}$: half-activation potential, E_{rev}: reversal potential, k: slope rate. $V_{0.5,act}$: half-activation potential of activation curve, $V_{0.5,inact}$: half-inactivation potential of inactivation curve. *P<0.05 versus Ctrl, unpaired t test.

Table S6. Summary of Cav1.2 channel electrophysiological characteristics of NG, GS or GS plus H-89-treated VSMCs

	$V_{0.5}$ (mV)	E_{rev} (mV)	k	n
NG	-2.14±1.73	55.49±1.72	-10.13±0.79	12
GS	-9.06±1.13**	49.49±1.44*	-7.39±0.65*	12
GS+H-89	-7.68±1.55*	51.51±1.55	-9.50±0.78	15

$V_{0.5}$: half-activation potential, E_{rev}: reversal potential, k: slope rate. *P<0.05, **P<0.01 versus NG-treated VSMCs, 1-way ANOVA followed by a Tukey's post hoc test.

Table S7. Summary of Cav1.2 channel electrophysiological characteristics of vehicle (Ctrl), MGO or MGO plus H-89-treated VSMCs

	$V_{0.5}$ (mV)	E_{rev} (mV)	k	n
Ctrl	-1.80±2.37	55.56±2.12	-10.95±1.01	8
MGO	-10.23±1.71*	54.86±1.98	-10.04±0.84	9
MGO+H-89	-9.69±1.75*	50.04±1.38	-11.37±0.80	9

$V_{0.5}$: half-activation potential, E_{rev}: reversal potential, k: slope rate. *P<0.05 versus vehicle- treated VSMCs, 1-way ANOVA followed by a Tukey's post hoc test.

Table S8. Summary of Cav1.2 channel electrophysiological characteristics of NT or Rbfox1 siRNA-treated VSMCs

	$V_{0.5}$ (mV)	E_{rev} (mV)	k	n
NT siRNA	-2.51±1.26	49.34±1.15	-8.21±0.66	11
Rbfox1 siRNA	-8.75±1.24**	47.84±1.36	-7.96±0.69	14

$V_{0.5}$: half-activation potential, E_{rev}: reversal potential, k: slope rate. **P<0.01 versus NT siRNAs-treated VSMCs, unpaired t test.

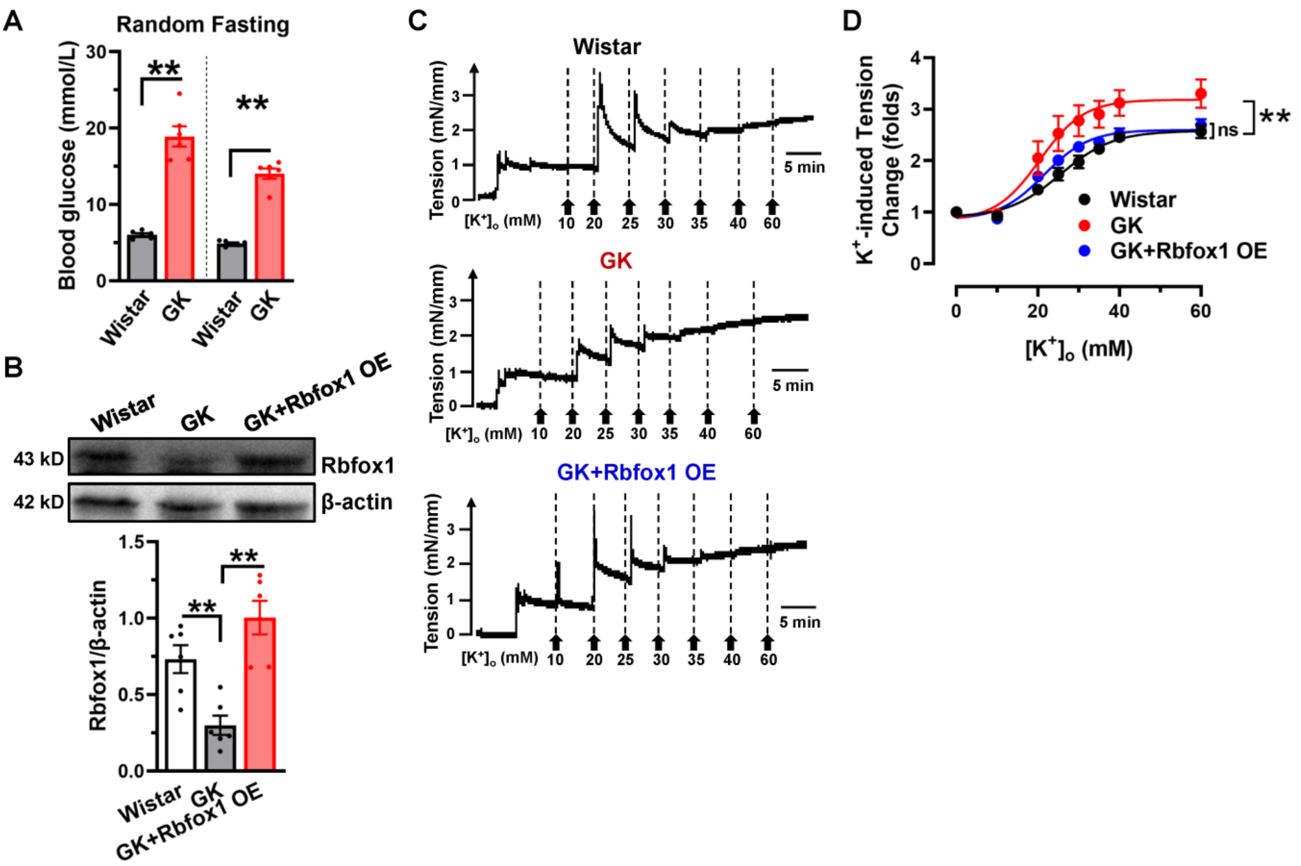

Figure S1. Overexpression of Rbfox1 in mesenteric arteries reduces vasoconstriction in Goto-Kakizaki (GK) rats. (**A**) Random and fasting blood glucose levels in the rats. (**B**) Expression of Rbfox1 was detected 3 days after treatment by Rbfox1 expression plasmids (OE) in mesenteric arteries from GK rats. β-actin served as an internal control. Band intensities were analyzed to show the relative expression of Rbfox1 and summarized as a bar chart. n=6 rats for each group, 1-way ANOVA followed by a Tukey's post hoc test. (**C**) Exemplary traces of mesenteric artery tension responding to increased KCl extracellular concentration (from 0 to 60 mmol/L KCl) using vascular myograph in Wistar, GK and Rbfox1 overexpressed GK rats. (**D**) Plots of concentration-tension relationship were represented and fitted with Boltzmann equation for differently-treated arteries. n=6 rats for each group. *P<0.05 vs Wistar rats, 2-way ANOVA followed by Sidak's multiple comparisons.

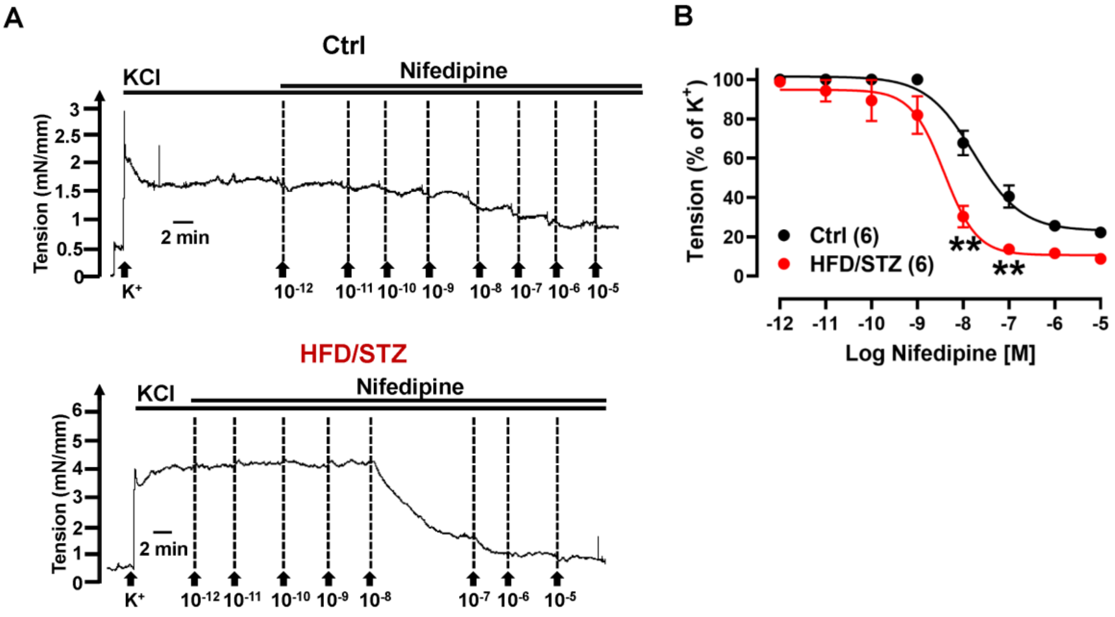

Figure S2. MAs from diabetic rats are more sensitive to nifedipine in comparison to control ones. (**A**) Arterial tension responding to different concentration of nifedipine under 60 mmol/L KCl bath solution was measured by vascular myography in control (Ctrl) or HFD/STZ-treated rats (one artery for each rat). (**B**) Plots of concentration-tension relationship were represented for the MAs from control or diabetic rats. ***P*<0.01 vs control rats, 2-way ANOVA followed by Sidak's multiple comparisons.

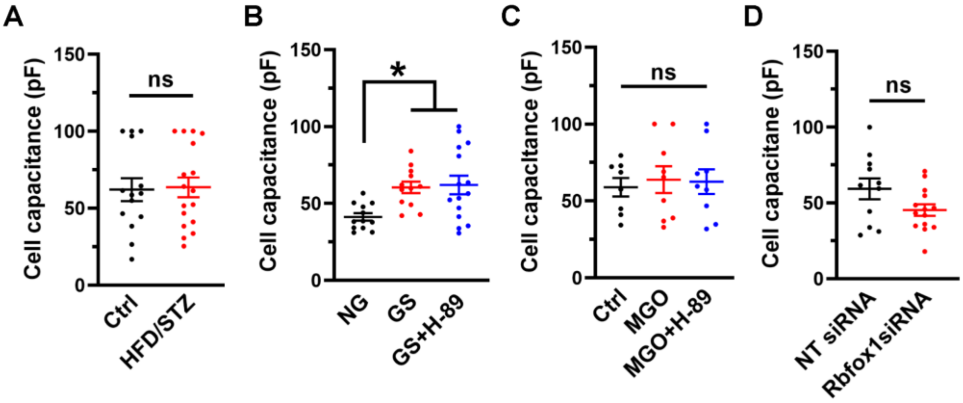

Figure S3. The cell capacitances (C_m) are measured in differentially-treated VSMCs. The C_m were shown as scatter plots in freshly isolated VSMCs of MAs from control or HFD/STZ-treated rats (**A**), in VSMCs treated with NG, GS or GS plus H-89 (**B**), in VSMCs treated with vehicle (Ctrl), MGO or MGO plus H-89 (**C**), and in NT or Rbfox1 siRNA-treated VSMCs (**D**). **P*<0.05, ns indicates no significant differences, unpaired *t* test or 1-way ANOVA followed by a Tukey's post hoc test.

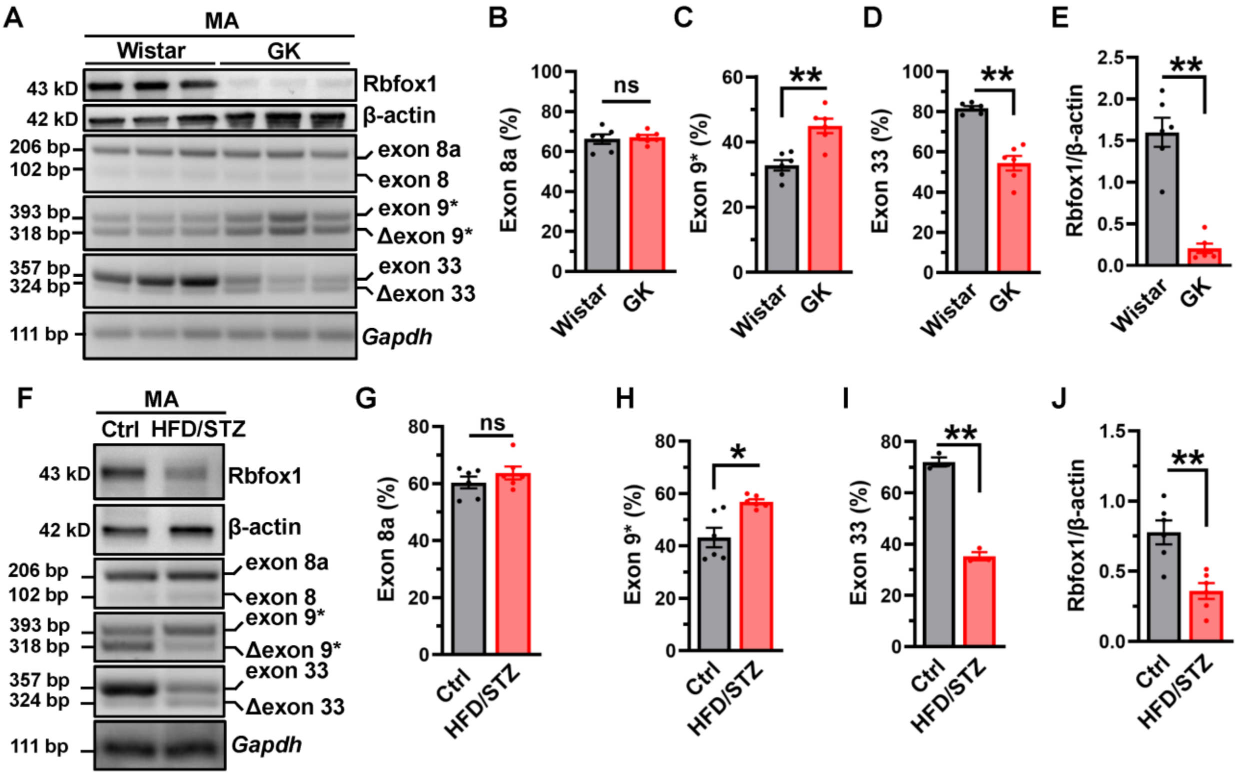

Figure S4. Cav1.2 channel is aberrantly spliced in the arteries from GK rats and non-hypertensive HFD/STZ rats. Mesenteric arteries (MAs) from Wistar or GK rats, and Ctrl or HFD/STZ rats were isolated to determine the expression level of Rbfox1 protein by Western blotting. The proportion of Cav1.2 with exon 8/8a, exon 9* or exon 33 were detected by RT-PCR. The relative Rbfox1 expression was normalized to β-actin after treatments **(A, F)**. The values for percent exon 8/8a **(B, G)**, exon 9* **(C, H)** or exon 33 inclusion **(D, I)** of Cav1.2 channels were presented as bar charts. Relative expression of Rbfox1 and summarized as a bar chart (**E, J**). n=3-6 rats for each group. ns indicates no significant differences, *P<0.05, **P<0.01 vs Wistar or Ctrl rats, unpaired t test.

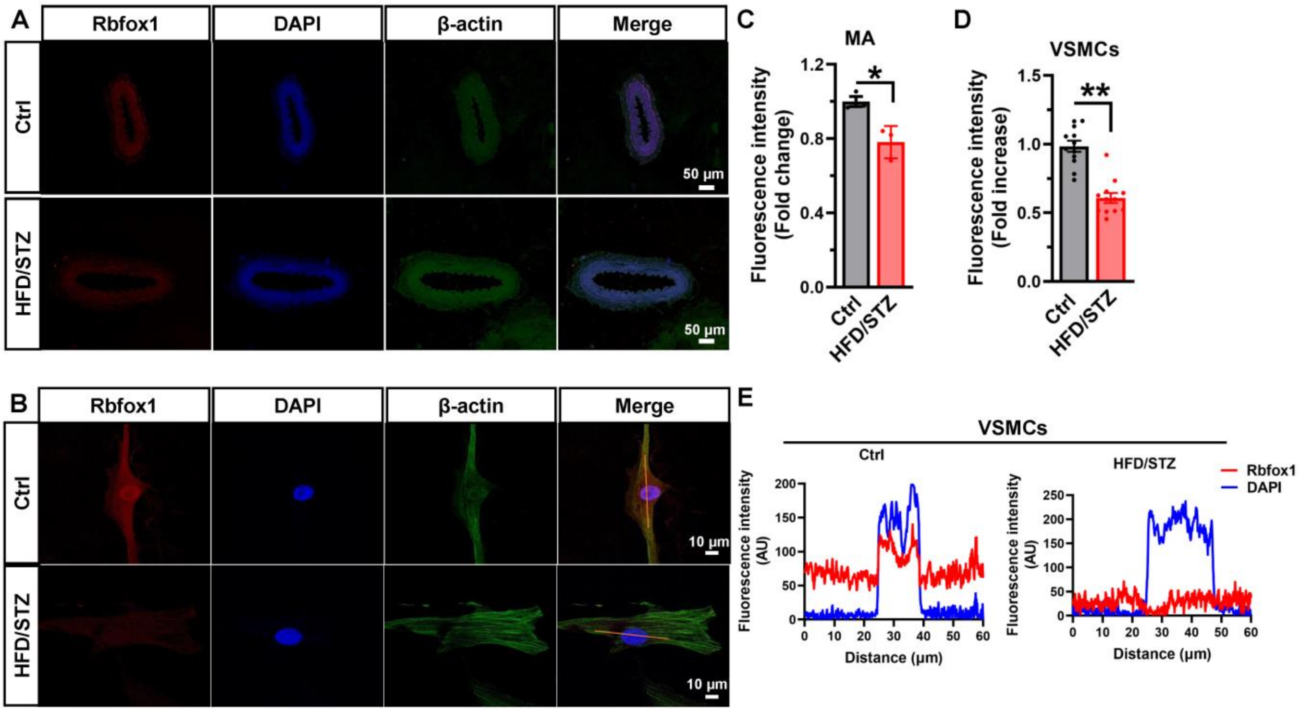

Figure S5. Rbfox1 is downregulated in the arteries/VSMCs from HFD/STZ rats. Immunofluorescence staining was used to detect the expression of Rbfox1 in MAs (**A**) or isolated VSMCs (**B**) from Ctrl and HFD/STZ rats. Fluorescence intensities were analyzed (**C&D)**. (**E**) Fluorescence intensity profiles for Rbfox1 (red) and DAPI (blue) along the yellow line drawn in the pictures, expressed as Arbitrary Unit (AU), in Ctrl and HFD/STZ rats. (n=3 arteries and 12 cells from 3 rats, respectively). **P*<0.05, ***P*<0.01 vs ctrl, unpaired *t* test.

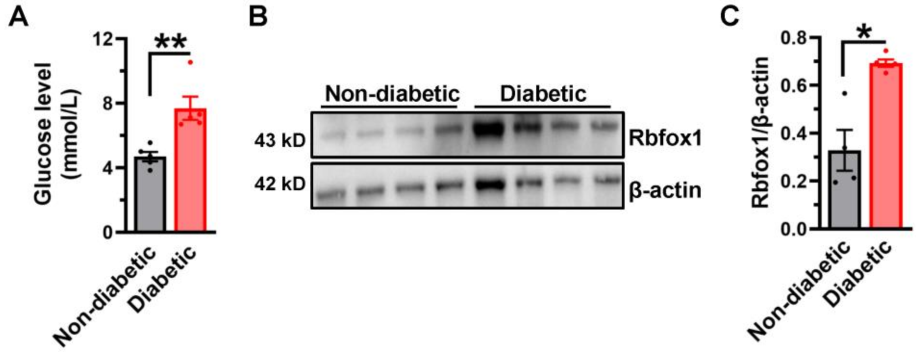

Figure S6. Rbfox1 is upregulated in the arteries from diabetic patients. (**A**) Blood glucose levels from the patients with or without diabetes. ***P*=0.0048 vs non-diabetic patients, unpaired *t* test. (**B**) Rbfox1 protein expression detected by Western blotting in diabetic versus nondiabetic cerebral arteries from the patients, β-actin was served as an internal control. (**C**) The relative Rbfox1 expression was normalized to β-actin, and presented as a bar chart. **P*=0.022 vs non-diabetic patients, unpaired *t* test with Welch's correction.

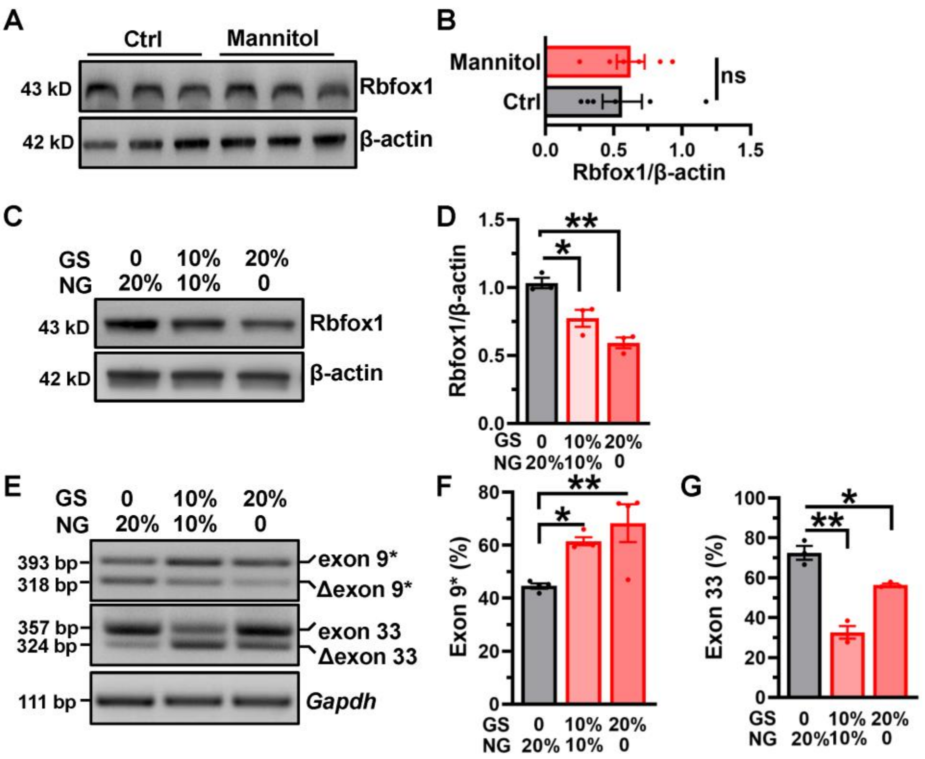

Figure S7. GS decreases Rbfox1 expression and regulates Ca$_V$1.2 alternative exons 9* and 33. (**A**) Mannitol (25 mmol/L) was used to treat isolated VSMCs for 48 hr. The expression of Rbfox1 was checked by Western blotting, and β-actin was detected as internal control. (**B**) The expression level of Rbfox1 was normalized to β-actin expression after mannitol treatment. *P*=0.7304, unpaired *t* test. (**C**) Rbfox1 expression was checked by Western blotting after treating with increasing concentration of GS. (**D**) The relative Rbfox1 expression levels were normalized by β-actin expression. (**E**) Expressions of Ca$_V$1.2 alternative exon 9* or exon 33 were checked by RT-PCR. The values for percent exon 9* (**F**) or exon 33 inclusion (**G**) were analyzed after treating with GS. **P*<0.05, ***P*<0.01 vs NG-treated VSMCs, 1-way ANOVA followed by a Tukey's post hoc test.

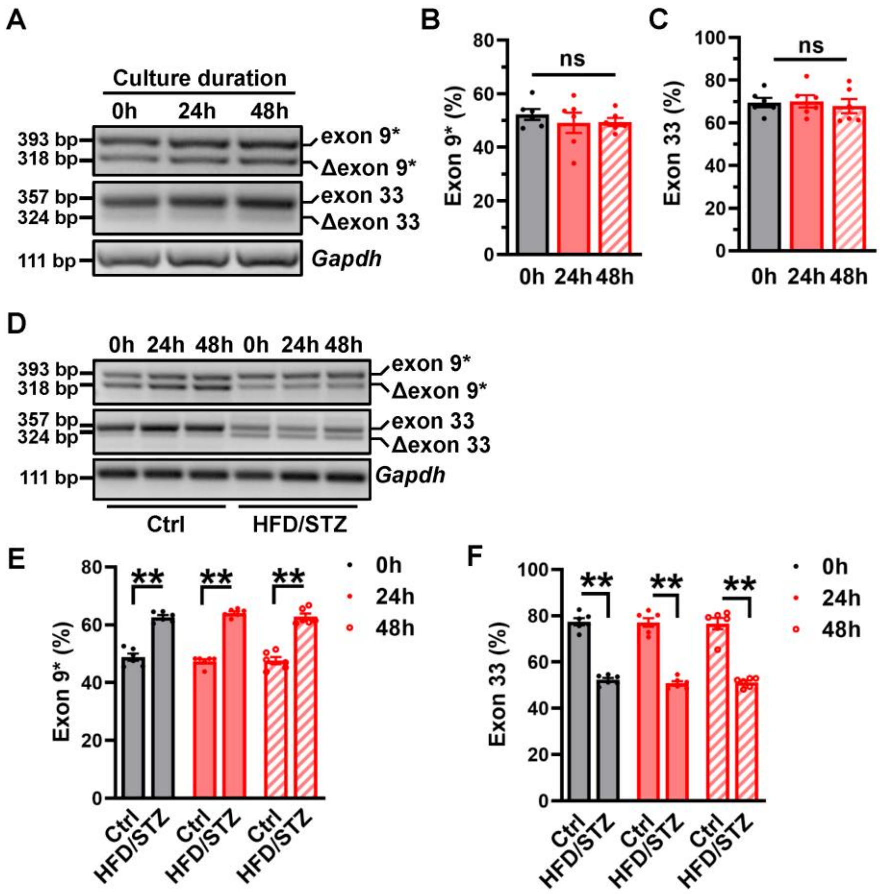

Figure S8. Cell culture does not alter Ca$_V$1.2 AS events in isolated VSMCs. (**A**) Freshly isolated VSMCs from rats (0 hr) were cultured with the medium for 24 or 48 hr. The proportion of Cav1.2 with alternative exon 9* (**B**) or exon 33 (**C**) were detected by RT-PCR. *Gapdh* mRNA was detected as loading control. ns indicates no significant differences, 1-way ANOVA followed by a Tukey's post hoc test. (**D**) Freshly isolated VSMCs from control or HFD/STZ rats (0 hr) were cultured with the medium for 24 or 48 hr. The proportion of Cav1.2 with alternative exon 9* (**B**) or exon 33 (**C**) were detected by RT-PCR. *Gapdh* mRNA was detected as loading control. ***P*<0.01 vs control rats, 2-way ANOVA followed by a Sidak's multiple comparisons.

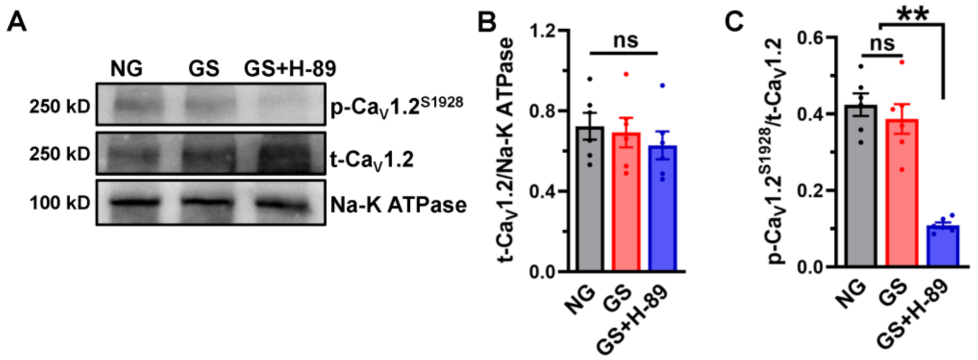

Figure S9. GS application does not increase Cav1.2 expression and S1928 phosphorylation in VSMCs. (**A**) VSMCs were treated with 20% NG, GS or GS plus 50 μmol/L PKA inhibitor H-89 for 48 hr and Western blotting were performed to detect the expression of total Cav1.2 (**B**) and S1928 phosphorylated Cav1.2 (**C**). $^{**}P<0.01$ vs GS. ns indicates no significant differences, 1-way ANOVA followed by a Tukey's post hoc test.

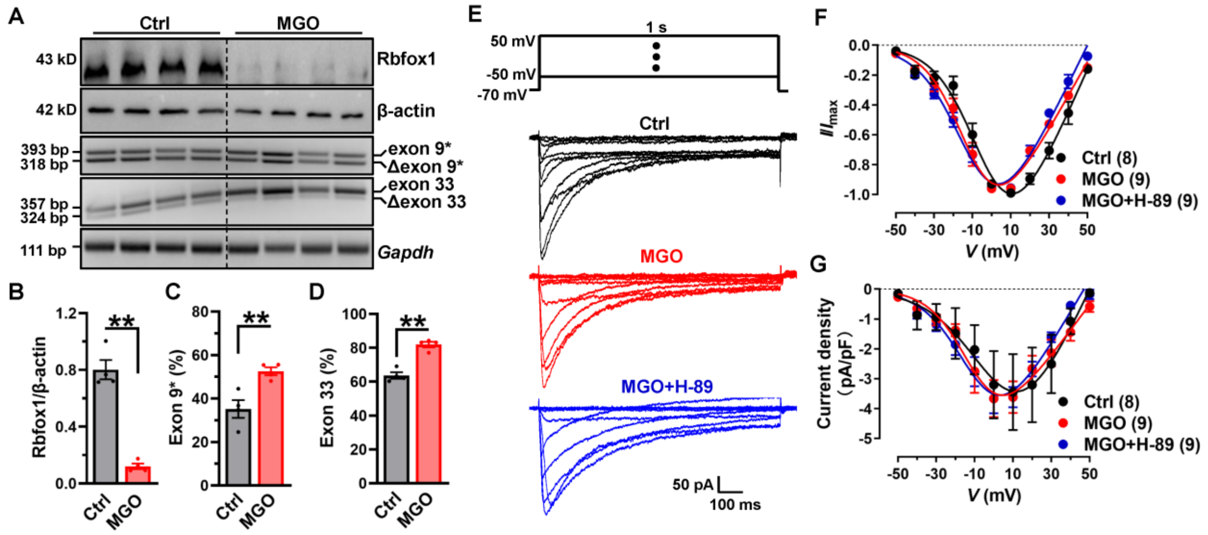

Figure S10. Methylglyoxal (MGO) induces decreased Rbfox1, aberrant Cav1.2 AS and hyperpolarization of current–voltage curve of Cav1.2 in VSMCs. (**A**) As one of main components in AGEs, MGO (500 μmol/L) was used to treat VSMCs for 48 hr and β-actin was detected as internal control. The proportion of Cav1.2 with alternative exon 9* or exon 33 were detected by RT-PCR. (**B**) The relative Rbfox1 expression level was presented in differently-treated VSMCs. The proportions of Cav1.2 with alternative exon 9* (**C**) or exon 33 (**D**) were analyzed in differently treated VSMCs. (**E**) Whole-cell currents in the Cav1.2 channel were recorded under different potentials, increasing from −50 to 50 mV (10-mV increase per step; *I–V* protocol) in isolated aortic VSMCs under 10 mmol/L Ba^{2+} bath solution. Additionally, 50 μmol/L PKA inhibitor H-89 was applied 2 hr before the Cav1.2 currents recording in MGO-treated VSMCs. (**F**) Plots of Cav1.2 current-voltage relationship (*I–V*) curve of VSMCs were analyzed after treating with vehicle (Ctrl), MGO or MGO plus H-89. (**G**) Current densities of Cav1.2 channel were calculated by currents divided by cell capacitance (*C*m) of the VSMCs.

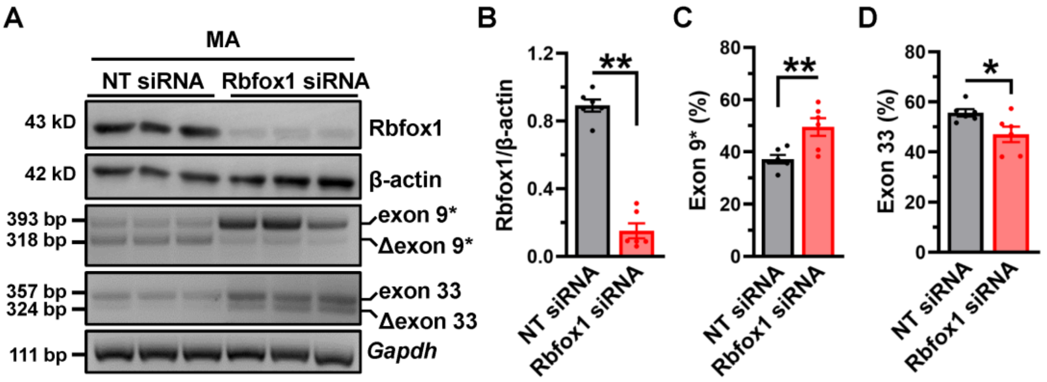

Figure S11. Rbfox1 dynamically regulates CaV1.2 alternative exons 9* and 33 in MAs. (**A**) NT or Rbfox1 siRNAs were transfected into isolated MAs using reversible permeabilization procedure. After 72 hr culture, Rbfox1 protein and Cav1.2 with alternative exon 9* or exon 33 were detected by Western blotting and RT-PCR, respectively. (**B**) The relative Rbfox1 expression was presented in MAs after different treatments. The values for percent exon 9* (**C**) or exon 33 inclusion (**D**) were presented as bar charts. **P*<0.05, ***P*<0.01 vs NT siRNA-treated MAs, unpaired *t* test.

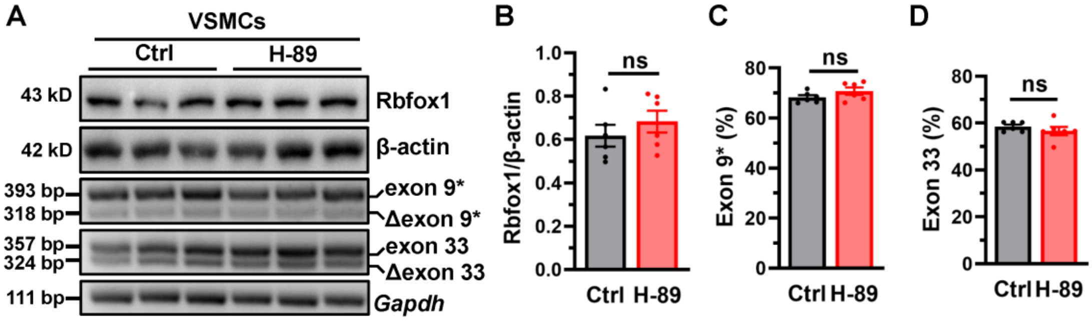

Figure S12. H-89 does not affect Rbfox1 expression and CaV1.2 AS events in NG-treated VSMCs. (**A**)Vehicle (Ctrl) or H-89 (50 µmol/L) was used to treat isolated VSMCs for 48 hr. The expression of Rbfox1 was checked by Western blotting, and the proportion of Cav1.2 with alternative exon 9* or exon 33 were detected by RT-PCR. (**B**) The relative Rbfox1 expression was normalized to β-actin after treatments. The values for percent exon 9* (**C**) or exon 33 inclusion (**D**) of Cav1.2 channels were presented as bar charts. ns indicates no significant differences, unpaired *t* test.

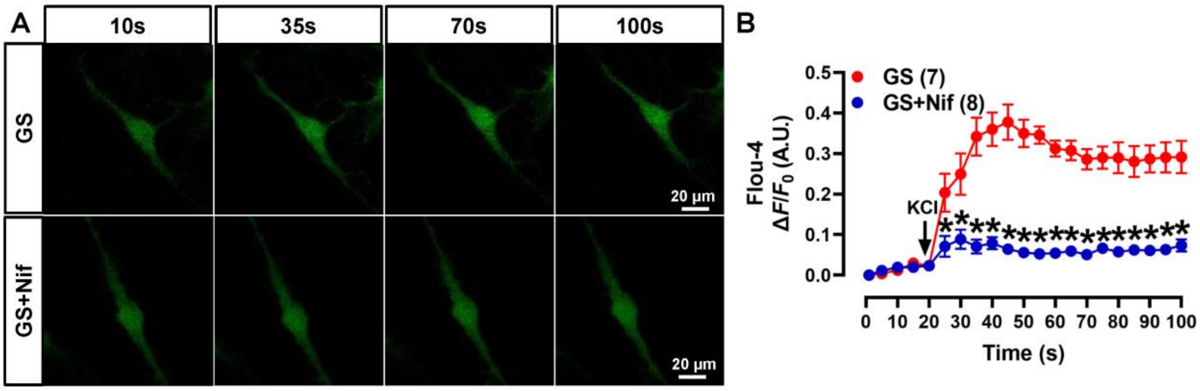

Figure S13. Application with nifedipine blocks GS-induced [Ca^{2+}]$_i$ elevation. (**A**) VSMCs were treated with 20% GS for 48 hr and 10^{-6} mol/L nifedipine (Nif) were added 10 min before [Ca^{2+}]$_i$ triggered by 60 mmol/L KCl, [Ca^{2+}]$_i$ was monitored by Ca^{2+} fluorescence indicator Fluo-4 AM. The fluorescent intensity was measured by time series scanning mode under a confocal microscope. (**B**) Δ[Ca^{2+}]$_i$ was presented as $\Delta F/F_0$ and shown as a line diagram, *P<0.01 vs GS-treated VSMCs, 2-way ANOVA followed by Sidak's multiple comparisons.

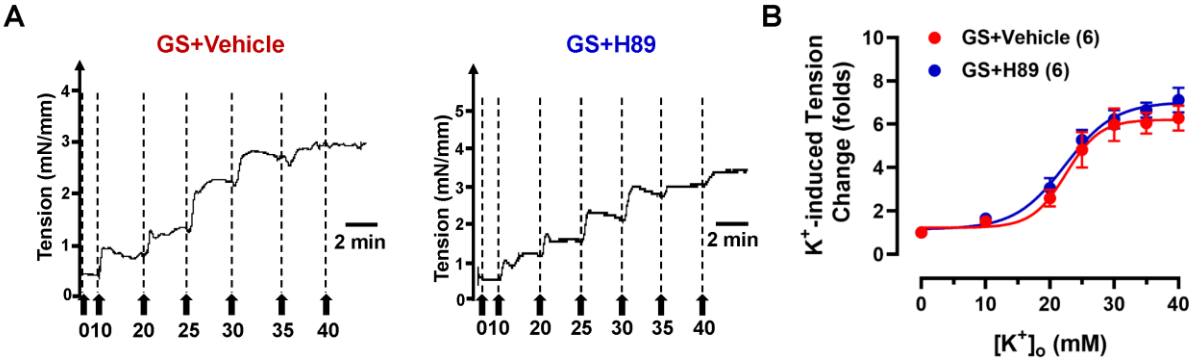

Figure S14. PKA inhibitor H-89 doesn't affect GS-induced vasoconstriction. (**A**) Isolated MAs were cultured with 20% GS with or without H-89 (50 μmol/L) for 48 hr, then the forced tension induced by increasing extracellular K^+ concentration was recorded by using vascular myograph. (**B**) Plots of concentration-tension relationship were represented and fitted with Boltzmann equation for differently-treated arteries. There were no significant statistical differences, 2-way ANOVA followed by Sidak's multiple comparisons.

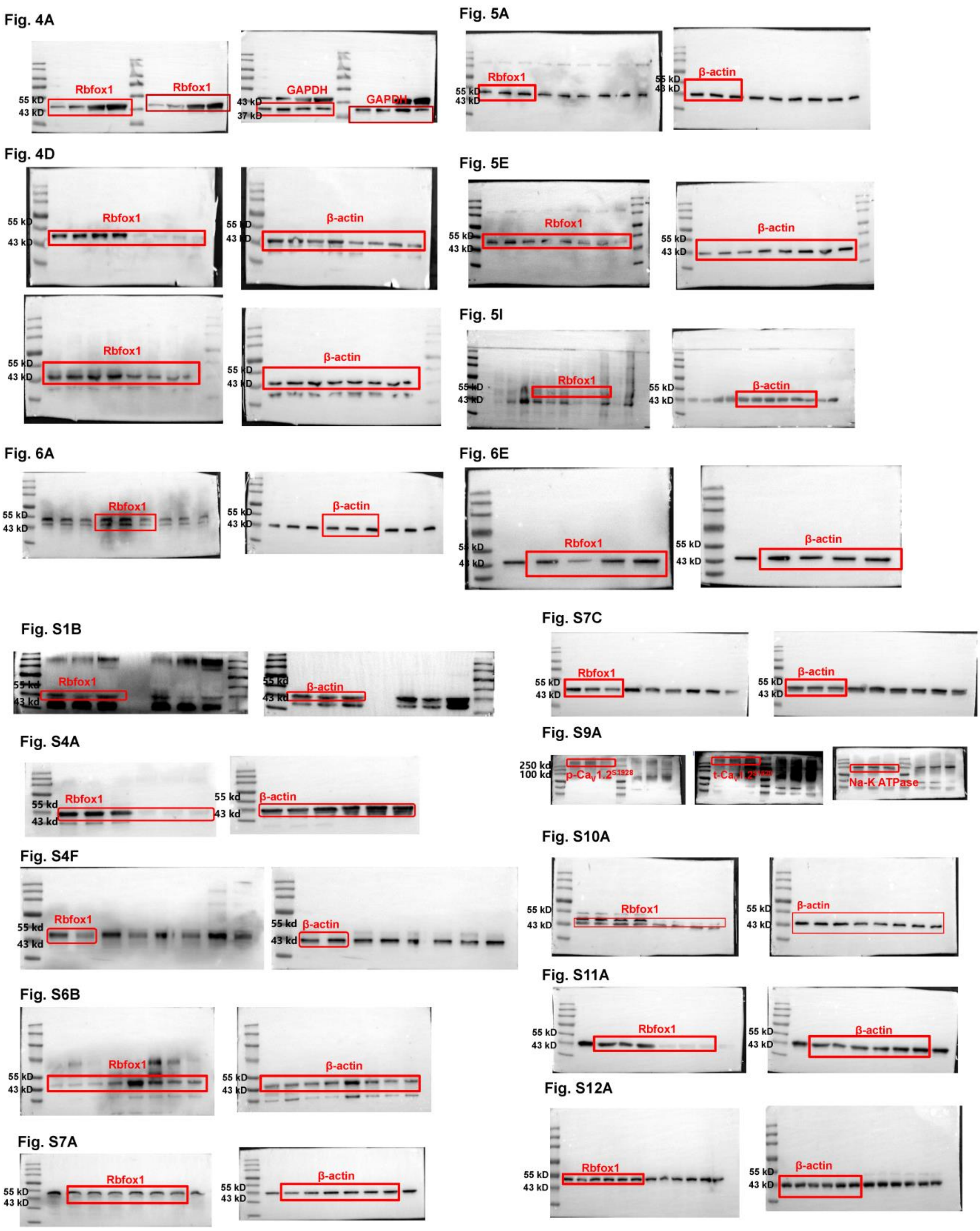

Figure S15. Uncropped Western blotting images.
